# Aberrant lipid metabolism in macrophages is associated with granuloma formation in sarcoidosis

**DOI:** 10.1164/rccm.202307-1273OC

**Published:** 2024-05-01

**Authors:** Clarice X. Lim, Anna Redl, Lisa Kleissl, Ram Vinay Pandey, Carolina Mayerhofer, Thomas El Jammal, Mario Mazic, Karine Gonzales, Nyamdelger Sukhbaatar, Thomas Krausgruber, Christoph Bock, Markus Hengstschläger, Alain Calender, Yves Pacheco, Georg Stary, Thomas Weichhart

**Affiliations:** 1 Center of Pathobiochemistry & Genetics, Institute of Medical Genetics, Medical University of Vienna, 1090 Vienna, Austria; 3 Department of Dermatology, Medical University of Vienna, 1090 Vienna, Austria; 4 CeMM Research Center for Molecular Medicine of the Austrian Academy of Sciences, 1090 Vienna, Austria; 5 Laboratory of Tissue Biology and Therapeutic Engineering, CNRS UMR5305, University Claude Bernard Lyon 1, IBCP, 69007 Lyon, France; 6 Medical University of Vienna, Institute of Artificial Intelligence, Center for Medical Data Science, Vienna, Austria; 7 Department of Genetics, Hospices Civils de Lyon, University Claude Bernard Lyon 1, 69500 Bron, France

**Keywords:** Sarcoidosis, Granuloma, lipid-laden, macrophages, non-Löfgren’s

## Abstract

**Rationale:**

Chronic sarcoidosis is a complex granulomatous disease with limited treatment options that can progress over time. Understanding the molecular pathways contributing to disease would aid in new therapeutic development.

**Objectives:**

To understand if macrophages from non-resolving chronic sarcoidosis patients are predisposed to macrophage aggregation and granuloma formation, and if modulation of the underlying molecular pathways influence sarcoidosis granuloma formation.

**Methods:**

Macrophages were cultivated *in vitro* from isolated peripheral blood CD14^+^ monocytes and evaluated for spontaneous aggregation. Transcriptomics analyses, phenotypic and drug inhibitory experiments were performed on these monocyte-derived macrophages. Human skin biopsies from sarcoidosis patients and a myeloid *Tsc2*-specific sarcoidosis mouse model were analyzed for validatory experiments.

**Measurements and Main Results:**

Monocyte-derived macrophages from chronic sarcoidosis patients spontaneously formed extensive granulomas *in vitro* compared to healthy controls. Transcriptomic analyses separated healthy and sarcoidosis macrophages and identified an enrichment in lipid metabolic processes. *In vitro* patient granulomas, sarcoidosis mouse model granulomas, and those directly analyzed from lesional patient skin expressed an aberrant lipid metabolism profile and contained increased neutral lipids. Conversely, a combination of statins and cholesterol-reducing agents reduced granuloma formation both *in vitro* and *in vivo* in a sarcoidosis mouse model.

**Conclusions:**

Together, our findings show that altered lipid metabolism in sarcoidosis macrophages is associated with its predisposition to granuloma formation and suggest cholesterol-reducing therapies as a treatment option in patients.

## Introduction

Sarcoidosis is a systemic inflammatory disease of unknown origin that is characterized by the presence of non-caseating granulomas in various organs, including the lungs, lymph nodes, skin, and heart (1). It is often self-limited or resolving upon treatment within 1-3 years in approximately two-thirds of patients (2). However, it can become chronic and worsen over time leaving the patient in a state of impaired quality of life (3), organ failure, and mortality in up to 8% of cases (4, 5).

Granulomas are compact aggregates of mononuclear phagocytes, which comprise mainly of macrophages (6). In sarcoidosis, CD4^+^ T cells are thought to play a central role in the disease, being driven by specific antigens and HLA-subtypes that are just beginning to be uncovered (7, 8). The occurrences of familial sarcoidosis cases with a heritability of about 39% suggest a contribution of genetic susceptibility in the pathogenesis of the disease (9). In mice, we previously showed that chronic activation of mTORC1 by deletion of its negative regulator *Tsc2* in macrophages alone leads to spontaneous development of a granulomatous phenotype in lungs and skin that resembles sarcoidosis (10). mTORC1 activation was also associated with disease progression in sarcoidosis patients (10). In addition, we have identified aberrant mTOR signaling as one of the top enriched pathways found in rare genetic variants identified in familial sarcoidosis patients (11). However, how mTORC1 reprograms macrophages to promote granuloma aggregation is currently unclear.

While insights have been made in the analyses of sarcoidosis patient material, such as peripheral blood monocytes or bronchoalveolar lavage (12–15), more studies focusing on chronic sarcoidosis patients as a distinct experimental sub-group are needed. Currently, it is believed that antigen-specific T cells are mainly responsible for granuloma formation in sarcoidosis. It is unclear if a genetic predisposition exists in sarcoidosis patients that would directly contribute to the granulomatous burden. While genetic manipulation of macrophages in mice recapitulates many features of granulomatous disease (10), whether macrophages are causative factors in human sarcoidosis is unknown (15).

Previously, we showed the transferability of the granuloma phenotype in the lungs of irradiated wild-type mice after reconstitution with *Tsc2* conditional knock-out bone marrow (10). This suggested that the cell type responsible for the formation of sarcoidosis granulomas is blood hematopoiesis-derived, likely a monocyte-derived macrophage. GM-CSF is a cytokine that is produced at increased quantities by epithelial cells, T cells, and myeloid cells during inflammation (16). It is also produced at higher amounts in sarcoidosis patient cells compared to healthy controls (17–20), as well as in sarcoidosis granulomas compared to tuberculosis granulomas or control tissues (21). GM-CSF induces monocyte differentiation into macrophages (16), enhances macrophage survival and promotes granuloma formation induced by microbial products (22, 23) or during sterile inflammation (24).

Here we describe a T cell-free *in vitro* granuloma assay, where we observed that GM-CSF stimulated monocyte-derived macrophages from chronic sarcoidosis patients formed bigger, and more spontaneous aggregates compared to monocyte-derived macrophages from healthy donors. These aggregating sarcoidosis macrophages were revealed by RNA sequencing analyses to have a dysregulated lipid metabolism, which we corroborated through single cell sequencing analyses and staining of neutral lipids in both sarcoidosis patient tissues and mouse model. Consequently, treatment with statins and cholesterol-reducing agents led to reductions in size and number of granulomas and reduced overall disease severity.

## Methods

The materials and methods used, including study design, patient sampling, monocyte-derived macrophage cell culture, *in vitro* granuloma quantification, the Tsc2^KO^ mouse model, flow cytometry analysis, immunohistochemistry and immunofluorescence, bulk RNA-seq and single-cell RNA-seq (scRNA-seq), treatments, bioinformatic and statistical analysis, are reported in the online supplement.

## Results

### Spontaneous granuloma formation of macrophages from chronic sarcoidosis patients

**Fig. 1:**
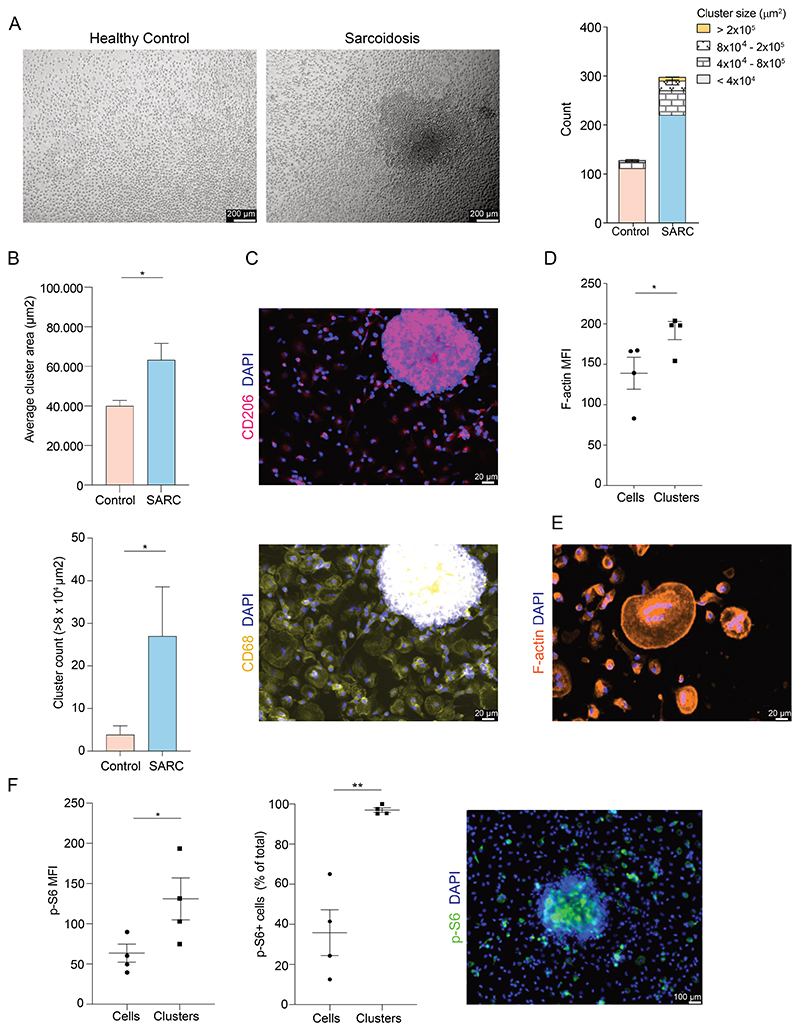
Chronic sarcoidosis patient monocyte-derived macrophages supplemented with GM-CSF form *in vitro* granuloma aggregates **(A)** Photomicrographs of day 6 monocyte-derived macrophages from chronic sarcoidosis patients and age-matched healthy controls (n=5) with corresponding quantification of macrophage aggregation according to cluster size. **(B)** Quantification of the average area and number of large and medium-sized macrophage aggregates/clusters that are bigger than 8x 10^4^ μm^2^ taken from Fig. 1A. **(C)** Representative immunofluorescent staining of a macrophage aggregate for CD206 (pink) and CD68 (yellow) expression. Nuclei stained with DAPI (blue). Staining performed for 4 patient samples. **(D)** Quantification of F-actin/Phalloidin expression (mean fluorescent intensity, MFI) in aggregating macrophage clusters vs remaining cells (n=4 patient samples). **(E)** Immunofluorescent staining of a macrophage aggregate for F-actin/Phalloidin (orange) expression. Nuclei stained with DAPI (blue). **(F)** Quantification of p-S6 expression (mean fluorescent intensity, MFI) found in aggregating macrophage clusters vs remaining non-clustering cells and frequency of p-S6^+^ cells amongst total cells (n=4 patient samples). Representative image of sarcoidosis macrophage aggregate stained with phospho-S6 ribosomal protein (p-S6) antibody.

From our previous results in mice, we hypothesized that macrophages from chronic sarcoidosis patients are intrinsically predisposed to granuloma formation. Thus, we recruited a cohort of patients with 14 chronic sarcoidosis and 10 age-matched controls with both pulmonary and/or skin involvement (Data file S1, Online Methods). These patients were also participants at the baseline of a clinical trial (EudraCT Number: 2017-004930-27). A series of assays were performed. In those assays, a minimum of 4 patient samples were used for each experiment (Data File S1, Online Methods). To test a potential direct contribution of macrophages in sarcoidosis granuloma formation, we obtained CD14^+^ blood monocytes and differentiated them into macrophages with GM-CSF for phenotypic observation and transcriptomic profiling (Fig. S1A). Interestingly, we observed large spontaneous aggregates of sarcoidosis monocyte-derived macrophages (MDM) after day 3 of culture, mimicking the formation of compact aggregates in early, ‘primitive’ granulomas (25). The sarcoidosis MDM formed bigger and more clusters compared to age-matched controls (Fig. 1A, 1B). The clusters consisted of CD68^+^ CD206^+^ expressing macrophages that were organized in compact structures (Fig. 1C, S1B), with tightly interdigitating cell membrane and increased cytoskeletal F-actin filaments compared to the remaining cells (Fig. 1D). Additionally, some of the aggregating macrophages also formed multi-nucleated cells (Fig. 1E). Proliferative macrophages play a role in the early stages of granuloma development (10, 23, 26). Similar to the lung granuloma clusters from the *Tsc2*-dependent sarcoidosis mouse model, we also found increased Ki-67-positive expression in the patient macrophage clusters (Fig S1C). Additionally, we noticed increased phosphorylation of the mTORC1 target S6 kinase and increased frequency of pS6-expressing cells in the macrophage clusters compared to the non-clustering cells (Fig. 1F, Fig. S1D). pS6 is a good marker for mTORC1 activation, responsible to mTOR inhibitors (rapamycin and everolimus) (10, 27).

### Enrichment of lipid metabolism and inflammatory response pathways among genes upregulated in sarcoidosis macrophages

**Fig. 2:**
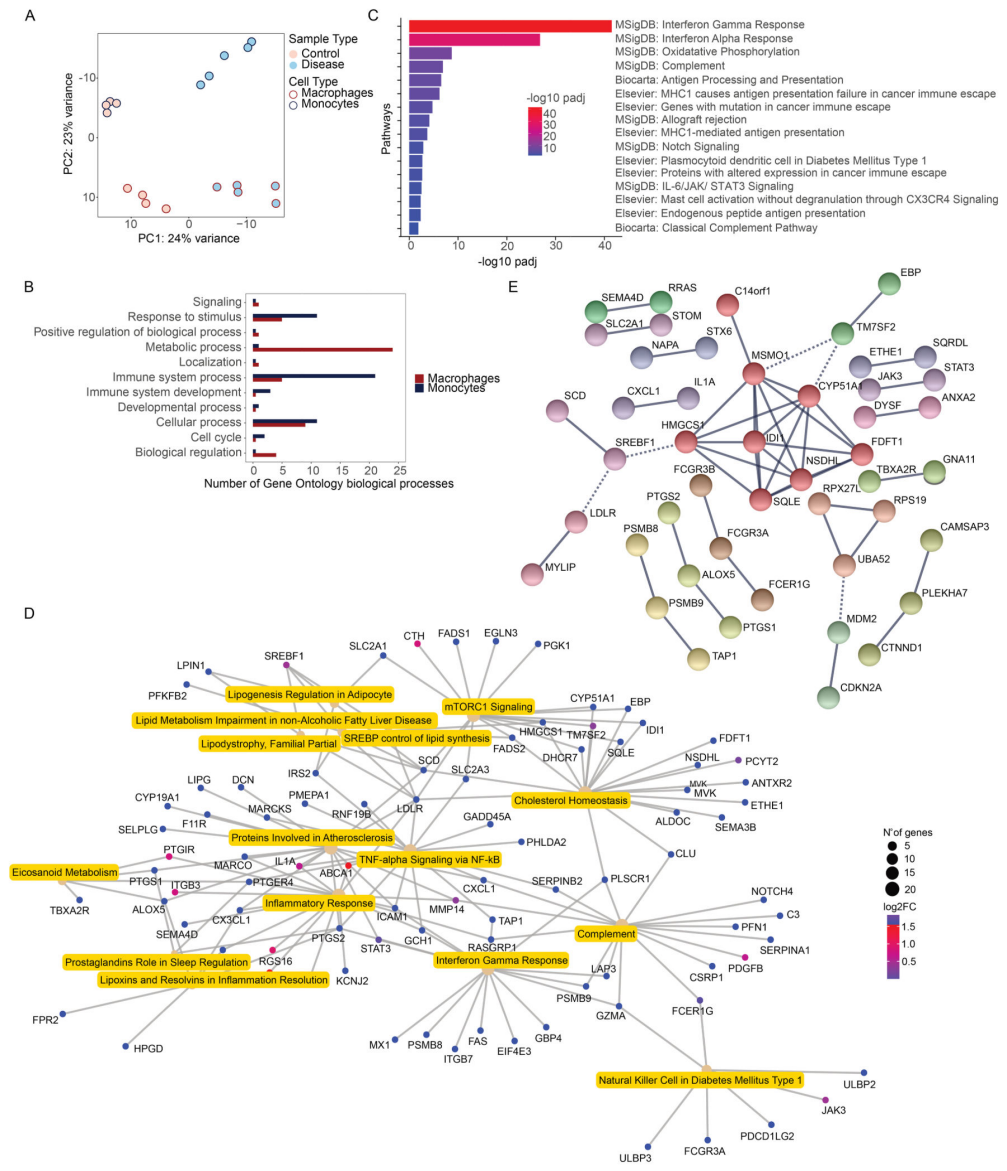
Bulk RNA sequencing analyses of chronic sarcoidosis monocytes and macrophages **(A)** Principal component analysis (PCA) plot of disease (sarcoidosis, n=5) and healthy control (n=4) monocytes and macrophages. **(B)** Bar plot of the number of enriched gene ontology (GO) sub-categories (x-axis) under each category (y-axis), based on genes differentially expressed in sarcoidosis monocytes versus control monocytes, and sarcoidosis macrophages versus control macrophages. **(C)** Bar plot of the pathways from MSigDB, Biocarta or Elsevier Pathway databases enriched in gene transcripts upregulated in sarcoidosis monocytes compared to healthy control monocytes (Enrichr pathway gene-set analyses, x-axis -log10 p-adjusted values). **(D)** Category-gene net plot of enriched pathways and genes from transcripts upregulated in sarcoidosis macrophages compared to healthy controls. Node size denote number of genes in pathway. Gene transcripts involved in individual pathways are represented extending from pathway categories. Color of gene transcript nodes represent its expression (log2 Fold change) relative to control. **(E)** Full STRING protein-protein interaction (PPI) analysis of corresponding proteins of genes upregulated in sarcoidosis macrophages. Nodes clustered using the Markov Cluster (MCL) Algorithm with inflation parameter =3. Cluster color labels clusters containing nodes with high interaction score. Inter-cluster edges are represented with dotted lines. Protein-protein enrichment value: 1.11e-16.

To identify pathways involved in *in vitro* granuloma formation, we performed bulk RNA sequencing on isolated CD14^+^ monocytes and GM-CSF induced MDMs obtained from 5 sarcoidosis patients and 4 non-sarcoidosis controls (Online Methods, Table S1, Data File S1). This resulted in 1,322 differentially regulated gene transcripts between sarcoidosis and control monocytes and 768 differentially regulated genes between sarcoidosis and control macrophages. The principal component analysis revealed that both sarcoidosis monocytes and macrophages clustered away from control non-sarcoidosis cells (Fig. 2A), indicating that sarcoidosis patient cells are transcriptionally distinct from non-sarcoidosis cells. Gene ontology analyses revealed an enrichment in “Response to stimulus” and “Immune system” biological processes in sarcoidosis monocytes compared to controls, and an enrichment in “Metabolic processes” and “Cellular processes” in sarcoidosis macrophages compared to control macrophages (Fig. 2B). Interestingly, 79 gene transcripts were upregulated both in sarcoidosis monocytes (Table S2) and macrophages (Table S3), and these genes were enriched in “Interferon gamma” and “STAT3 Signaling” pathways (Fig. S2A), with the inclusion of both STAT3 and JAK3 gene transcripts (Table S4). Over-representation gene set analyses of differentially regulated genes in sarcoidosis monocytes revealed that genes upregulated in sarcoidosis monocytes were enriched in “Interferon gamma”, “Interferon alpha”, “Oxidative phosphorylation”, “Antigen processing and presentation”, “Complement”, and “IL-6/JAK/STAT3” pathways (Fig. 2C, Table S5, Suppl. Fig 2B-2C); while genes downregulated in sarcoidosis monocytes were over-represented in a molecular signature found on the apical surface of epithelial cells. No significant enriched pathways were found from genes downregulated in sarcoidosis macrophages compared to healthy control macrophages.

From the genes upregulated in sarcoidosis macrophages, we found an enrichment of pathways related to lipid metabolism, such as “Cholesterol Homeostasis”, “SREBP control of lipid synthesis”, “Lipodystrophy, familial partial”, “Lipoxins and Resolvins in inflammation resolution”, “lipid metabolism impairment in non-alcoholic fatty liver disease”, and “Lipogenesis regulation in adipocyte” (from here on termed lipid metabolism cluster). Interestingly, the genes upregulated in sarcoidosis macrophages were also enriched in the pathway “mTORC1 Signaling”, and “Interferon Gamma Response” (Fig 2D, Table S6). We also performed STRING protein-protein interaction analyses and plotted out the predicted protein network based on all the genes upregulated in sarcoidosis macrophages compared to healthy controls (Fig. 2E). Again, we found similar genes in the lipid metabolism cluster pathways namely *SREBF1, HMGCS1, CYP51A1, NSDHL, SQLE, IDI1, MSMO1, TM7SF2, EBP, SCD, LDLR*, and *MYLIP* that were highlighted in the analysis. Again, many of those genes overlapped with those in the “mTORC1 Signaling” pathway (Suppl. Fig. 2D-2E, Table S6).

### Aberrant lipid metabolism in macrophages is responsible for *in vitro* granuloma formation

**Fig. 3:**
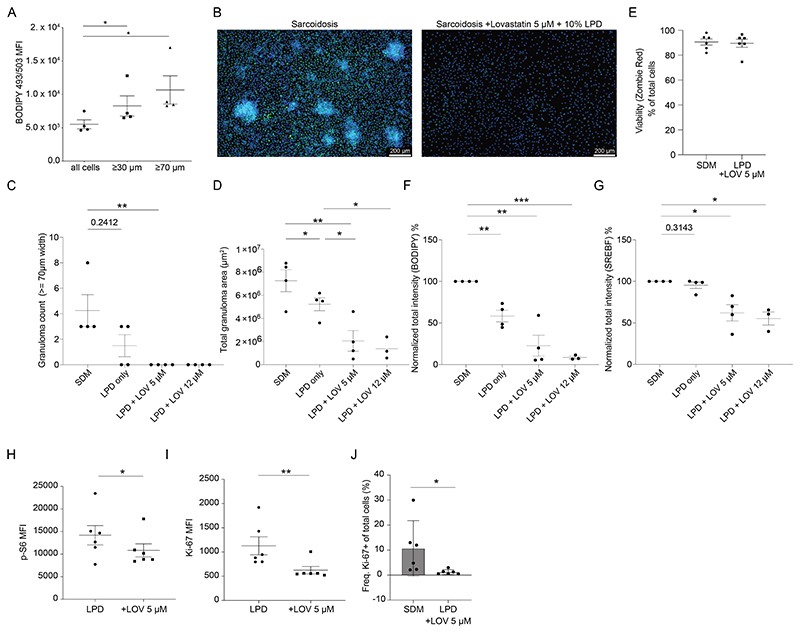
Lipid metabolism pathways play a role in the formation of sarcoidosis *in vitro* granulomas **(A)** BODIPY 493/503 neutral lipid expression in sarcoidosis macrophage clusters (n= 4 patient samples) => 30 microns wide, and => 70 microns μm wide versus all cells (identified by DAPI nuclei staining) in sarcoidosis monocyte-derived macrophage culture. **(B)** Representative images of sarcoidosis macrophages at day 6 of culture with GM-CSF after 3-day treatment with standard media with 10% fetal calf serum and control DMSO diluent (SDM) or 10% lipoprotein-deficient serum and 5 μM Lovastatin. Green spots: BODIPY 493/503 neutral lipids, DAPI (blue). **(C)** Number of large macrophage aggregates (>=70μm width) after 3 days of treatment with standard media with 10% fetal calf serum and control DMSO diluent (SDM), or 10% Lipoprotein deficient media alone (LPD), or 5μM Lovastatin or 12μM Lovastatin and 10% lipoprotein-deficient serum. Two-tailed paired T-tests carried out. **(D)** Quantification of *in vitro* granuloma size (area in m^2^) after 3 days of treatment with standard media with 10% fetal calf serum and control DMSO diluent (SDM), or 10% Lipoprotein deficient media alone (LPD), or 5μM Lovastatin or 12μM Lovastatin and 10% lipoprotein-deficient serum. Two-tailed paired T-tests carried out. Significant Fixed effect (type III) p < 0.01 (mixed effect analysis). **(E)** Treated and control-treated sarcoidosis macrophages (treatment as described in Fig. 3B) were stained with Zombie Red (Biolegend) dye to assess viability by flow cytometry (n= 6 patient samples). **(F)** Total intensity (integral density) of BODIPY 493/503 neutral lipid expression in sarcoidosis macrophages treated with standard media with 10% fetal calf serum and control DMSO diluent (SDM), or 10% Lipoprotein deficient media alone (LPD), or 5μM Lovastatin or 12 μM Lovastatin and 10% lipoprotein-deficient serum was measured and values normalized to the SDM macrophage condition and expressed as a percentage (%). Two-tailed paired T-tests carried out. Significant Fixed effect (type III) p < 0.01 (mixed effect analysis). **(G)** Total intensity (integral intensity/density) of SREBF1 in sarcoidosis macrophages treated with standard media with 10% fetal calf serum and control DMSO diluent (SDM), or 10% Lipoprotein deficient media alone (LPD), or 5μM Lovastatin or 12μM Lovastatin and 10% lipoprotein-deficient serum was measured and values normalized to the SDM macrophage condition and expressed as a percentage (%). Two-tailed paired T-tests carried out. Significant Fixed effect (type III) p < 0.05 (mixed effect analysis). **(H)** Phospho-S6 (pS6) expression in sarcoidosis macrophages treated with 10% lipoprotein-deficient serum and control DMSO or 5μM Lovastatin and 10% lipoprotein-deficient serum determined by flow cytometry (n=6 patient samples). **(I)** Ki-67 expression in sarcoidosis macrophages treated with 10% lipoprotein-deficient serum and control DMSO or 5μM Lovastatin and 10% lipoprotein-deficient serum determined by flow cytometry (n= 6 patient samples). **(J)** Frequency of ki-67+ stained cells determined by flow cytometry after treatment with control DMSO diluent in standard media containing 10% fetal calf serum or 5μM Lovastatin and 10% lipoprotein-deficient serum (n= 6 patient samples).

Given the striking enrichment of cholesterol metabolism genes and pathways in sarcoidosis macrophages, we wondered if this is connected to enhanced granuloma formation *in vitro*. Cholesterol is the most abundant neutral lipid species in cells, which can be increased by synthesis or uptake. Thus, we measured neutral lipid content by BODIPY 493/503 (28)staining in sarcoidosis macrophages that formed clusters (=> 30μm or 70μm) versus non-cluster forming cells. Indeed, cluster-forming granulomatous cells contained more neutral lipids compared to non-cluster forming cells (Fig. 3A, S3A). Hence, we decided to interfere with the lipid/cholesterol state of sarcoidosis macrophages after they formed *in vitro* granulomas. Statins are a class of cholesterol lowering drugs that inhibit HMG-CoA reductase, an enzyme that is important in the production of cholesterol, fatty acids and lipids from acetyl-CoA (29). The combined treatment with both Lovastatin and lipoprotein-deficient serum reduced the number and size of *in vitro* granulomas formed by the sarcoidosis macrophages (Fig 3B, 3C, 3D) while maintaining viability (Fig. 3E). Single treatment of lipoprotein-deficient serum was sufficient in reducing granuloma area (Fig. 3D) and smaller (=>30μM) granulomas (Suppl Fig 3B) but was not able to reduce numbers of larger (=>70μM) granulomas (Fig. 3C). In cultures with lipoprotein-deficient serum neutral lipid content was reduced upon treatment with increasing concentrations of Lovastatin (Fig. 3F). *SREBF1*/SREBP1a/c is a transcription factor regulating lipid and fatty acid synthesis (30), which was predicted to be upregulated in sarcoidosis macrophages by RNA-seq (Fig. 2D). Indeed, we found high SREBF1a/c protein expression in clusters that was reduced upon treatment with the combination of Lovastatin and lipoprotein-deficient serum (Fig. 3G). Single treatments of either Lovastatin or lipoprotein-deficient serum did not affect lipid levels and SREBF1 expression (Fig. S3C, Fig. 3G). The reduction in number and size of the granuloma clusters after the combined treatment with Lovastatin and lipoprotein deficient serum was also associated with reduced S6 phosphorylation/mTORC1 activation (Fig. 3H), and reduced proliferation of sarcoidosis macrophages (Fig. 3I, 3J).

Our RNA sequencing data revealed the JAK/STAT pathways to be upregulated in both sarcoidosis monocytes and macrophages (Table 2 and 3). Hence, we treated the macrophage granuloma aggregates of sarcoidosis patients with Tofacitinib, a JAK2/3 inhibitor that was shown to be effective in treating patients with longstanding cutaneous sarcoidosis (19, 31). However, treatment with Tofacitinib did not reduce the number or size of the *in vitro* sarcoidosis MDM clusters (Fig. S3D, S3E). Accordingly, neutral lipid content in the macrophages clusters was unaltered upon Tofacitinib treatment (Fig. S3F).

### Increased neutral lipids in sarcoidosis granulomas in human skin biopsies

**Fig. 4:**
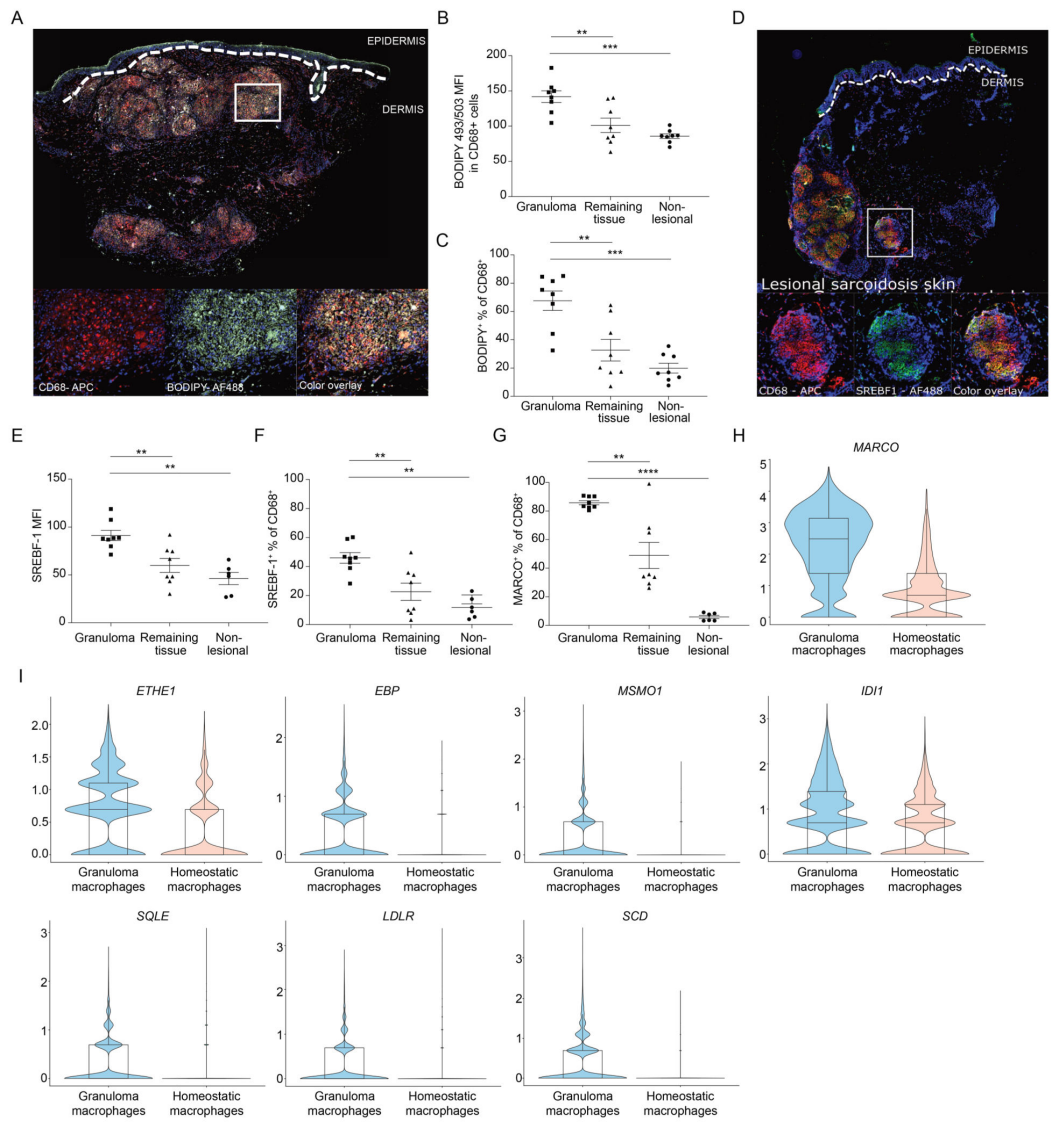
Lesional skin granulomas from sarcoidosis patients contain increased neutral lipids and an aberrant lipid metabolism profile **(A)** Representative immunofluorescence images of lesional skin stained with CD68, BODIPY 493/503 or DAPI from a chronic sarcoidosis patient (n=4 patient samples). **(B)** Quantification of BODIPY 493/503 neutral lipid expression in lesional skin granuloma vs remaining tissue in lesional skin vs non-lesional skin (n=4 in duplicates). **(C)** Frequency of BODIPY 493/503 neutral lipid^+^ CD68^+^ macrophages in sarcoidosis lesional skin granuloma vs remaining tissue in lesional skin vs non-lesional skin. **(D)** Representative images of sarcoidosis lesional skin stained with CD68, SREBF1 and DAPI (n=4 patient samples). **(E)** SREBF1 expression in CD68^+^ macrophages found in sarcoidosis lesional skin, remaining non-granulomatous tissue in lesional skin (n=4) and non-lesional skin (n=3). **(F)** Frequency of SREBF1^+^ CD68^+^ macrophages in sarcoidosis lesional skin, remaining tissue in lesional skin (n=4) and non-lesional skin (n=3). **(G)** Frequency of MARCO^+^ CD68^+^ macrophages in sarcoidosis lesional skin, lesional skin remaining tissue (n=4) and non-lesional skin (n=3). **(H)** Violin plots showing expression of MARCO in granuloma-associated (GA) macrophages and homeostatic macrophages (Cluster 0 and Cluster 1 from Krausgruber et. al., 2023). **(I)** Violin plots showing expression of lipid metabolism pathways genes (*ETHE1, EBP, MSMO1, IDI1, SQLE, LDLR* and *SCD*) in granuloma-associated (GA) macrophages and homeostatic macrophages (Cluster 0 and Cluster 1 from Krausgruber et. al., 2023).

To corroborate our observation of increased neutral lipids found in spontaneously aggregating sarcoidosis macrophages cultivated *in vitro* with GM-CSF, we obtained lesional skin biopsy samples from 6-8 chronic sarcoidosis patients with cutaneous and pulmonary involvement. We detected increased neutral lipid content in CD68^+^ macrophages from the granulomatous areas compared to the interjacent non-granulomatous areas of lesional skin biopsies and compared to macrophages from non-lesional skin biopsies of the same patients using both BODIPY 493/503 (Fig. 4A-4B, Fig S4A, B) and LipidTox stainings (Fig S4C). Frequencies of BODIPY-positive CD68^+^ macrophages were also higher in the granuloma areas of the lesional skin compared to the remaining tissue as well as to non-lesional skin (Fig. 4C). These findings show that lipid-loaded macrophages are an important cell population in cutaneous granulomas of sarcoidosis patients. Similar to the *in vitro* granuloma clusters, sarcoidosis skin granuloma macrophages highly expressed SREBF1a/c compared to non-granulomatous and non-lesional skin macrophages (Fig. 4D-4E). Moreover, the frequency of SREBF1a/c-positive macrophages was also increased (Fig. 4F). MARCO is a class A scavenger receptor that is associated with lipid uptake (32, 33) and proinflammatory host defense to bacterial pathogens (34), and upregulated in sarcoidosis macrophages compared to controls (Table 3). Accordingly, more MARCO expressing macrophages were found in lesional skin granulomas compared to the remaining tissue and non-lesional skin (Fig. 4G). Additionally, spatial transcriptomics and scRNA-seq data of human sarcoidosis lesional and non-lesional skin revealed increased *SREBF1* and *SREBF2* expression in sarcoidosis granulomas (20). Further analyses of data also revealed higher expression of *MARCO* (Fig. 4H) and lipid metabolism-related genes in sarcoidosis patient macrophages (Fig. 2E; *ETHE1, EBP, MSMO1, IDI1, SQLE, LDLR, SCD)* from cutaneous sarcoidosis granulomas compared to homeostatic macrophages in non-granuloma regions (Fig. 4I). Altogether, human sarcoidosis skin granulomas are characterized by the presence of lipid-laden CD68^+^ macrophages with aberrant lipid metabolism.

### Aberrant lipid metabolism and increased neutral lipids in sarcoidosis mouse model macrophages

**Fig. 5:**
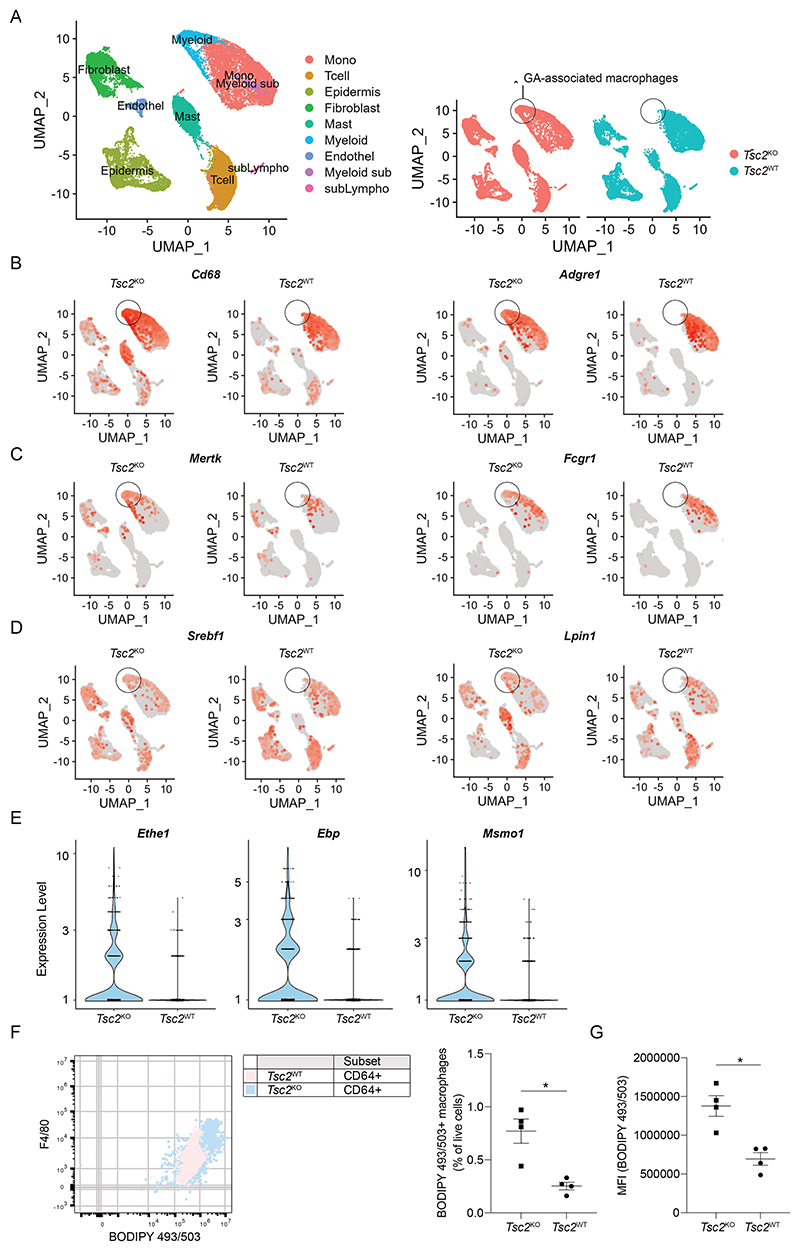
Macrophages from skin of sarcoidosis model mice display an aberrant lipid metabolism profile **(A)** UMAP of scRNA-seq transcriptome profiles from swollen paw and tail skin of female age-matched *Tsc2*^floxed/floxed^ CD11c-Cre (*Tsc2*^KO^) sarcoidosis model mice and *Tsc2*^floxed/floxed^ control (*Tsc2*^WT^) littermates between ages 36-41 weeks old (n=3 each). **(B)** UMAP from (Fig. 5A) annotated with expression of macrophage markers *Cd68* and *Adgre1* (F4/80). **(C)** UMAP from (Fig. 5A) annotated with expression of macrophage markers Mertk and Fcgr1 (CD64). **(D)** UMAP from (Fig. 5A) annotated with expression of lipid metabolism cluster genes – *Srebf1*, and *Lpin1*. **(E)** Violin plots showing expression of lipid metabolism associated genes (*Ethe1, Ebp, Msmo1)* in *Tsc2*^KO^ sarcoidosis mouse skin macrophages compared to *Tsc2*^WT^ control macrophages. Student’s t-test was performed. p= <0.001 for all three plots. **(F)** Frequency of BODIPY 493/503^+^ macrophages (gated on CD11b, activated macrophage marker Mac-2, CD64 and F4/80) amongst all live 7AAD-cells in the skin of sarcoidosis model mice (*Tsc2*^KO^) vs control *Tsc2*^WT^ mice (33-36 weeks old male mice, n=4 each) and representative dot plot showing population of F4/80, BODIPY 493/503 expressing cells amongst the gated macrophages. **(G)** Expression of BODIPY 493/503 neutral lipids in skin macrophages of sarcoidosis model mice (*Tsc2*^KO^) vs control *Tsc2*^WT^ mice (33-36 weeks old male mice, n=4 each).

Because interfering with lipid metabolism reduced *in vitro* granulomas from sarcoidosis patients, we wondered if such a treatment would functionally reduce disease severity and granuloma load *in vivo*. To do so, we utilized our previously described sarcoidosis mouse model that recapitulates both chronic and progressive disease state (10). To understand if the sarcoidosis model mice presented with similar aberrant lipid metabolism, we analyzed scRNA-seq profiles of CD45^+^ immune cells from the skin of *Tsc2*^floxed/floxed^ (termed *Tsc2*^WT^) and *Tsc2*^floxed/floxed^;CD11c-cre (*Tsc2*^KO^) mice and identified the major immune cell populations in the skin (Fig. 5A, left panel). Interestingly, we found a CD68^+^ F4/80^+^ (*Adgre1*) macrophage population in the skin of *Tsc2*^KO^ sarcoidosis mice (Fig. 5B) that was not present in *Tsc2*^WT^ mice (Fig. 5A, right panel). This population here referred to as “Granuloma-associated macrophages” (GA-macrophages) also expressed classic macrophage markers such as Mertk, and monocyte-macrophage marker CD64/Fcgr1 (Fig. 5A-C) (35). Mouse GA-macrophages expressed similar lipid metabolism related genes identified from patient macrophages like *Srebf1, Lpin1* (Fig. 5D), *Hmgcs1, Nsdhl, Dhcr7, Cyp51* (mouse analog of *CYP51A1*), *Aldoc, Mvk* (Fig. S5A). Additionally, genes that we previously identified to be higher expressed in sarcoidosis patient lesional skin macrophages, were also higher expressed in GA-associated macrophages in Tsc2^KO^ mice compared to macrophages of control (Tsc2^WT^) mice: *Ethe1, Ebp, Msmo1* (Fig. 5E), Aldoc, Mvk, *Sqle*, and *Ldlr* (Fig. S5B). Likewise, *Marco* was higher expressed in *Tsc2*^KO^ macrophages compared to *Tsc2*^WT^ macrophages, but in another macrophage sub-population (Fig. S5C). These alterations corresponded to a visible skin phenotype with increased thickening of the skin, most noticeable at the mouse paws and the tail (10). Importantly, in Tsc2^KO^ mice we found increased numbers of BODIPY 493/503-positive GA-macrophages (F4/80^+^ CD11b^+^ CD64^+^ Mac-2^+^) (Fig. 5F), which also showed increased accumulation of neutral lipids (Fig. 5G).

### Reduced disease severity in sarcoidosis mice treated with cholesterol-free diet and atorvastatin

**Fig. 6:**
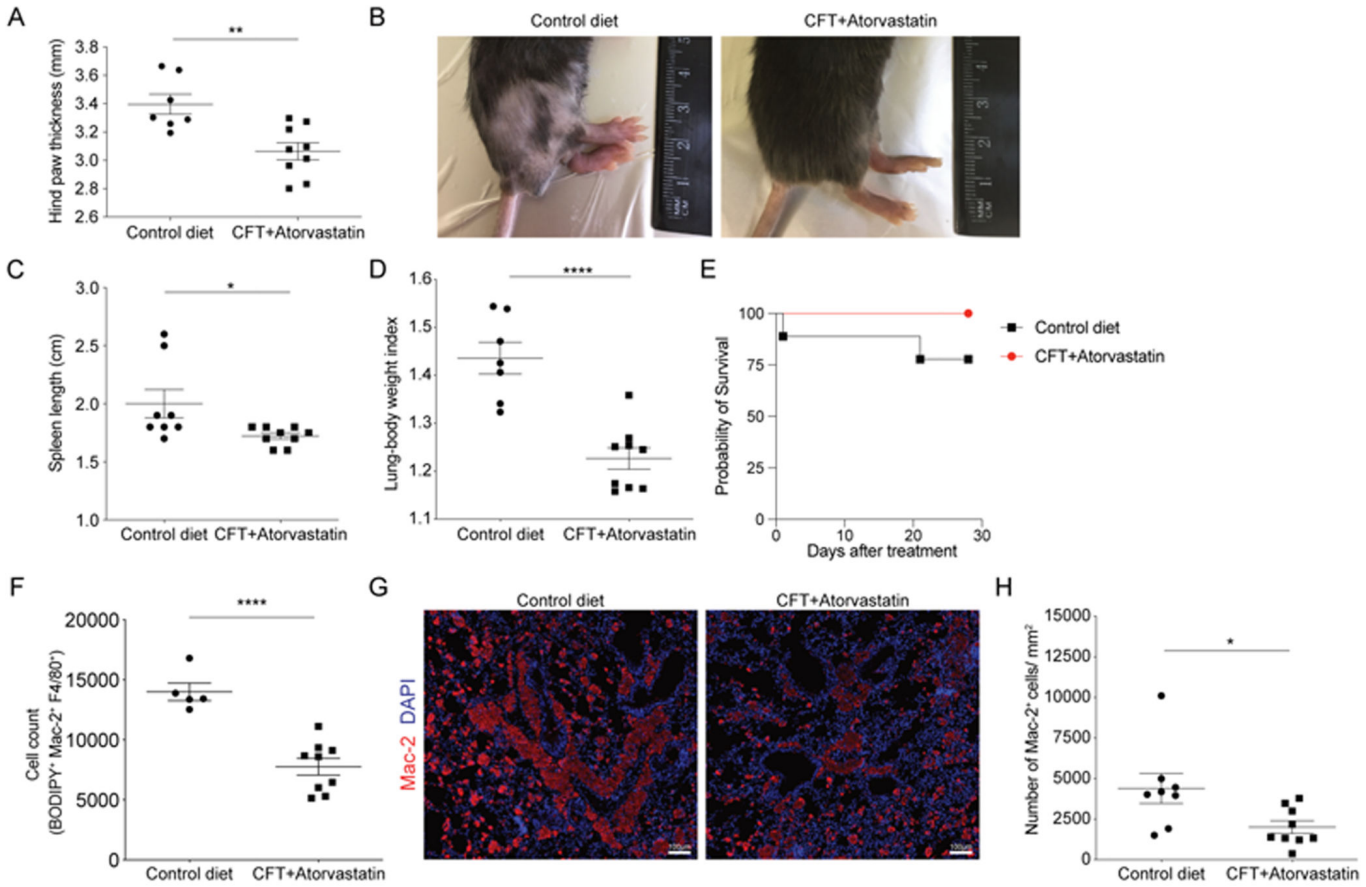
Reduction of disease severity after mice with severe sarcoidosis were treated with statin and cholesterol-deficient diet **(A)** Hind paw thickness of *TSC2*^KO^ sarcoidosis mice after treatment with cholesterol-deficient diet (CFT) and Atorvastatin/ Lipitor or control diet (n=9) and control DMSO diluent (n=7). **(B)** Representative image of sarcoidosis mice after treatment with cholesterol-deficient diet (CFT) and Atorvastatin/ Lipitor or control diet and control DMSO diluent. **(C)** Measurement of sarcoidosis mouse spleen length after treatment with cholesterol-deficient diet (CFT) and Atorvastatin/ Lipitor or control diet and control DMSO diluent. **(D)** Lung weight to body weight ratio index of sarcoidosis mice after treatment with cholesterol-deficient diet (CFT) and Atorvastatin/ Lipitor or control diet and control DMSO diluent. **(E)** Kaplan-Meier survival curve of severe sarcoidosis mice (weeks old) after 28 days of treatment with cholesterol-deficient diet (CFT) and Atorvastatin/ Lipitor (n=9) and or control diet and control DMSO diluent (n=9). **(F)** Number of BODIPY 493/503 positive F4/80^+^ Mac-2^+^ macrophages in lungs of sarcoidosis mice treated with cholesterol-deficient diet (CFT) and Atorvastatin/ Lipitor treated mice or control diet, and control DMSO diluent. **(G)** Photomicrographs of Mac-2-stained pulmonary granulomas of sarcoidosis mice treated with cholesterol-deficient diet (CFT) and Atorvastatin/ Lipitor treated mice or control diet, control DMSO diluent. **(H)** Number of Mac-2^+^ granulomas/ mm^2^ of lung in lungs of sarcoidosis mice treated with cholesterol-deficient diet (CFT) and Atorvastatin/ Lipitor treated mice or control diet, control DMSO diluent.

After validating that macrophages from the *Tsc2*^KO^ mouse model resemble sarcoidosis patient *in vitro* granulomas and cutaneous lesional granulomatous macrophages, we treated aged mice that have a severe sarcoidosis phenotype with lipid/cholesterol-modifying regimens. *Tsc2*^KO^ mice develop spontaneous sarcoid-like granulomas in their lungs and skin that progresses and becomes more severe as the mice age (10). As mentioned above, the mice presented with symptoms such as swelling of the paws, loss of hair, splenomegaly, and increased lung weight index, denoting increased pulmonary inflammation. After 28 days of treatment with a cholesterol-free diet and atorvastatin, treated mice showed reduced swelling of the hind paw, less balding (Fig. 6A and 6B), a reduction in splenomegaly (Fig. 6C), reduced lung weight and lung weight index (Fig. 6D, Fig. S6A). The mice showed increased survival at a severe stage of disease (Fig. 6E) with no significant changes in weight between the treatment groups (Fig S6B). Skin and lungs of Tsc2^KO^ mice also contained more lipid-laden macrophages with increased neutral lipid content (Fig. S6C) compared to Tsc2^WT^ mice. We observed a reduction in total lipid-laden macrophages in the lungs of treated mice compared to controls (Fig. 6F) as well as reduced myeloid CD11b expression in the lung (Fig. S6D). Importantly, immunofluorescence staining revealed reduced numbers of Mac-2 positive granulomas (36) in the lungs of treated mice compared to controls (Fig. 6G, 6H). These results indicate that reprogramming the lipid metabolic status of *Tsc2*^KO^ mice through statins and reduction in cholesterol-intake promotes a reduction in overall granuloma disease severity (Fig. 6A-H).

Together, our results suggest that macrophages are key players in granuloma formation, and a distinct transcriptomic profile leading to dysregulated lipid metabolism in macrophages is associated with the initiation of granuloma formation during persistent sarcoidosis.

## Discussion

Sarcoidosis is a complex granulomatous disease of yet unknown etiology (37). Although antigen-specific CD4 T cells are thought to play a primary role in sarcoidosis granuloma formation (7), it is not clear if macrophages from sarcoidosis patients are intrinsically prone to form granulomas. Current *in vitro* models of sarcoidosis granulomas utilize mostly peripheral blood mononuclear cells (PBMC) from human patients and require either microbial or antigenic stimulation for the cells to form *in vitro* cell aggregates resembling granulomas (38, 39). In these models, it is not clear whether T-cells previously induced by antigenic triggers, are responsible for the clustering phenotype *in vitro* or whether macrophages harbor alterations that directly promote and initiate the granulomatous burden. In this study, we hypothesized that sarcoidosis macrophages are intrinsically prone to form granulomas and sought to culture them *in vitro* from sorted CD14^+^ monocytes without the presence of T cells. Through supplementation with GM-CSF alone, differentiated macrophages from sarcoidosis patients clustered spontaneously *in vitro* and formed organized aggregates without the need for stimulation with beads or microbial antigen. This observation supports work from others who showed that monocytes are able to develop *in vitro* into cells that make up granulomatous inflammation (6, 40) and highlights the potential role of macrophages as a primary cell type in an inflammatory setting involved in the initiation of a sarcoid granuloma. This study also supports the use of GM-CSF in sarcoidosis granuloma models, previously found to promote granuloma formation in pulmonary histiocytosis (41), Schistosoma (22) and zymocel-induced hepatic granulomas (23).

In this study, we identified several enriched pathways upregulated in sarcoidosis macrophages, categorized broadly as inflammatory response-related pathways such as the interferon gamma response, TNF-alpha signaling pathway and mTORC1 signaling pathway, These pathways are globally in line with the molecular pathways targeted by drugs used in the clinics, such as the use of anti-inflammatory glucocorticoids, secondary therapeutic agents indicated for clinical use such as TNF-alpha inhibitors (Infliximab and adalimumab), Tofacitinib (a JAK inhibitor), as well as mTOR inhibitors (19, 37, 42).

Notably, our pathway analyses data from sarcoidosis *in vitro* granulomas unexpectedly indicated that lipid metabolism pathways in sarcoidosis macrophages are dysregulated. Thereafter, we were able to show that both sarcoidosis *in vitro* granulomas and cutaneous lesional sarcoidosis granulomas contained increased neutral lipids and upregulated expression of genes involved in lipid/cholesterol metabolic pathways such as SREBF-1a/c or MARCO. Interestingly, many of these upregulated genes are also involved in the mTORC1 signaling pathway. Using our chronic Tsc2^KO^ model, we found that the mice contain a granulomatous macrophage population not found in control mice that was also characterized by the expression of lipid metabolism-associated genes. Furthermore, treatment with statin and cholesterol-reducing agents (lipoprotein deficient serum or cholesterol-deficient diet) *in vitro* and *in vivo* reduced the number and size of *in vitro* granulomas, SREBF-1a/c expression, mTORC1 activation, and improved disease severity and granuloma count and size in the Tsc2^KO^ model. Interestingly, genes upregulated in bronchoalveolar lavage from patients with reduced lung function in the GRADS study cohort also revealed enrichment in SCAP/SREBP transcriptional control of cholesterol and fatty acid biosynthesis (43). In the GRADS and the validation cohort from Freiburg, bronchoalveolar samples with an increased macrophage fraction were also associated with PI3K/AKT signaling -upstream of mTORC1 (43).

An altered circulating lipid profile with increased triglycerides and lowered high density lipoprotein cholesterol has been observed in sarcoidosis patients (44, 45), with an indication for increased risk of atherosclerosis (46, 47). Indeed, in a small clinical trial where twenty-four atorvastatin-treated patients (80 mg/day) and placebo controls were treated for a year, the mild-to-moderate sarcoidosis patients that were treated with atorvastatin had a promising reduction in flare risk/relapse, defined as a “physiological deterioration in pulmonary function due to worsened pulmonary inflammation” (48). In our study, 6 out of 14 patients (Data File S1) were on statins. Our finding of dysregulated lipid metabolism in the granulomas of these patients was consistent for patients on statins and those not taking statins. Interestingly, statin-treated patient RP#12 had less *in vitro* granulomas compared to similarly chronic patients (disease duration 2-7 years) who were not statin-treated or treated with a lower dose of statins. However, our sample size is too small to find associations. The dose of atorvastatin used in the treatment of the mice would roughly convert to a human equivalent of 150 mg per day (49).

One limitation of our study is that our results are based on a small cohort of patients. Nevertheless, we observed a distinct lipid-laden macrophage profile in sarcoidosis granulomas validated by scRNA-seq analyses of both patient and mouse model cells, and histochemical stainings of lesional patient granulomas. Hence, based on the results from our study, treating patients with statins together with a low cholesterol diet may provide clinical benefit. In addition, our macrophage-centric *in vitro* model cannot recapitulate the complexity of a true *in vivo* granuloma or other PBMC models. Nevertheless, our study suggests the importance of the macrophages as a cell-type important in sarcoidosis granuloma formation and disease progression that could be used for testing of new therapeutic drugs targeting macrophages in chronic sarcoidosis.

## Supplementary Material

Online Methods

Supp. Fig.

## One Sentence Summary

Lipid metabolic processes in macrophages are associated with granuloma formation in chronic sarcoidosis.
