# Supplementary material for: Aberrant lipid metabolism in macrophages is associated with granuloma formation in sarcoidosis
Source: Am J Respir Crit Care Med. Author manuscript; Available in PMC 2025 Mar 21. (PMC7617514; doi:10.1164/rccm.202307-1273OC)
Supplement: Online Methods [file EMS203765-supplement-Online_Methods.docx]

**Aberrant Lipid Metabolism in Macrophages Is Associated with Granuloma Formation in Sarcoidosis**

Clarice X. Lim, Anna Redl, Lisa Kleissl, Ram Vinay Pandey, Carolina Mayerhofer, Thomas El Jammal, Mario Mazic, Karine Gonzales, Nyamdelger Sukhbaatar, Thomas Krausgruber, Christoph Bock, Markus Hengstschläger, Alain Calender, Yves Pacheco, Georg Stary, Thomas Weichhart

**ONLINE DATA SUPPLEMENT**

**LIST OF SUPPLEMENTARY MATERIALS**

Fig. S1: Spontaneous aggregation of chronic sarcoidosis monocyte-derived macrophages supplemented with GM-CSF

Fig. S2: Pathway gene-set analyses of genes upregulated in sarcoidosis patient cells

Fig. S3: Increased neutral lipids in sarcoidosis granulomas

Fig. S4. Aberrant lipid metabolism profile in CD68^+^, F4/80^+^ myeloid cells found in skin of sarcoidosis model mice

Fig. S5. Statin and cholesterol-deficient diet treatment of mice with severe sarcoidosis reduces granuloma inflammation

Data file S1: Chronic sarcoidosis study cohort and list of assays performed

Table S1: Chronic sarcoidosis cohort included in RNA sequencing analyses

Table S2: 79 upregulated genes in sarcoidosis monocytes compared to healthy controls

Table S3: 79 upregulated genes in sarcoidosis macrophages compared to healthy controls

Table S4. Pathway gene set analyses from genes upregulated in sarcoidosis monocytes compared to healthy control monocytes

Table S5. Pathway gene set analyses from genes upregulated in sarcoidosis macrophages compared to healthy control macrophages

Table S6. Pathway gene set analyses from 79 genes upregulated in both sarcoidosis monocytes and sarcoidosis macrophages

Online Methods

**SUPPLEMENTARY FIGURE LEGENDS**

**Fig. S1.** Spontaneous aggregation of chronic sarcoidosis monocyte-derived macrophages supplemented with GM-CSF

**(A)** Schema diagram of patient cohort and methods used in the study

**(B)** Quantification of CD206 and CD68 expression (mean fluorescent intensity, MFI) in aggregating macrophage clusters vs remaining non-clustering cells (n=4 patient samples).

**(C)** Quantification of Ki-67 expression (mean fluorescent intensity, MFI) found in aggregating macrophage clusters vs remaining non-clustering cells and frequency of Ki-67^+^ cells amongst total cells (n=3 patient samples).

(**D**) Control staining for DAPI only of the same image as in Figure 1F (left) and rabbit IgG – AF488 as isotype control for phosphor-S6 – AF488 in one monocyte derived macrophage cluster (right).

**Fig. S2.** Pathway gene-set analyses of genes upregulated in sarcoidosis patient cells

**(A)** Bar plot of the pathways from MSigDB, Biocarta or Elsevier Pathway databases enriched in the gene transcripts upregulated in both sarcoidosis monocytes and sarcoidosis macrophages compared to healthy control (Enrichr pathway gene-set analyses, x-axis -log10 p-adjusted values).

**(B-C)** Category-gene net plot of enriched pathways and genes from transcripts only upregulated in sarcoidosis monocytes compared to healthy controls. Node size denote number of genes in pathway. Gene transcripts involved in individual pathways are represented extending from pathway categories. Color of gene transcript nodes represent its expression (log2 Fold change) relative to control.

**(D)** Enrich-plot heat maps of inflammation-associated pathways and genes (HGNC gene symbol) upregulated in sarcoidosis macrophages compared to healthy control.

**(E)** Enrich-plot heat maps of metabolism-associated pathways and genes (HGNC gene symbol) upregulated in sarcoidosis macrophages compared to healthy control.

**Fig. S3.** Increased neutral lipids in sarcoidosis granulomas *in vitro*

**(A)** Representative photomicrograph of *in vitro* sarcoidosis granuloma stained with DAPI, BODIPY 493/503, and overlay of both (n= 4 patient samples).

**(B)** Number of small macrophage aggregates (>=30 μm width) after 3 days of treatment with standard media with 10% fetal calf serum and control DMSO diluent (SDM), or 10% Lipoprotein deficient media alone (LPD), or 5μM Lovastatin or 12 μM Lovastatin and 10% lipoprotein-deficient serum.

(**C**) Total intensity (integral intensity/density) of SREBF1 in sarcoidosis macrophages was measured and values normalized to the SDM macrophage condition and expressed as a percentage (%) The cells were treated for 3 days with: standard media with 10% fetal calf serum and control DMSO diluent (SDM), or 5μM Lovastatin alone in standard media with 10% fetal calf serum, or with 10% Lipoprotein deficient media alone (LPD), or 5μM Lovastatin or 12 μM Lovastatin and 10% lipoprotein-deficient serum was measured and values normalized to the SDM macrophage condition and expressed as a percentage (%).

**(D)** Quantification of *in vitro* sarcoidosis granuloma count after 3 days of treatment with 400 nM, 1000 nM Tofacitinib or control DMSO diluent in standard media with 10% fetal calf serum (n= 4 patient samples).

**(E)** Quantification of *in vitro* sarcoidosis granuloma size after 3 days of treatment with 400 nM, 1000 nM Tofacitinib or control DMSO diluent in standard media with 10% fetal calf serum.

**(E)** Normalized BODIPY 493/503 neutral lipid expression in sarcoidosis *in vitro* granulomas treated with 400 nM, 1000 nM Tofacitinib or control DMSO diluent in standard media with 10% fetal calf serum.

**Fig. S4.** Increased neutral lipids in skin sarcoidosis granulomas *in vivo*

(**A**) Matched representation of Figure 4A showing lipid droplets in sarcoidosis granuloma. Macrophages are visualized by the presence of CD68 (red) and neutral lipids using the BODIPY 493/503 dye (light green). DAPI (nuclei) in blue.

(**B**) DAPI single staining in one lesional sarcoidosis skin section as control for BODIPY 493/503 staining (left) and corresponding H&E staining to Figure 4A.

**(C)** Quantification of LipidTox neutral lipid expression and frequency of neutral lipid^+^ CD68^+^ macrophages in chronic sarcoidosis patient lesional skin granuloma vs remaining tissue in lesional skin vs non-lesional skin (n= 6 patients).

(**D**) Immunofluorescence staining for mouse IgG1-AF488 in lesional sarcoidosis skin as control for SREBF1-AF488 (left) and H&E staining of one matched skin section to Fig 4D (right).

**Figure S5** Aberrant lipid metabolism profile in CD68^+^, F4/80^+^ myeloid cells found in skin of sarcoidosis model mice

**(A)** UMAP of scRNA-seq transcriptome profiles of paw and tail skin of *Tsc2*^floxed/floxed^ CD11c-Cre (*Tsc2*^KO^) sarcoidosis model mice and *Tsc2*^floxed/floxed^ control (*Tsc2*^WT^) mice annotated with expression of lipid metabolism cluster genes: *Hmgcs1, Nsdhl, Dhcr7, Cyp51* (mouse analog of CYP51A1), *Aldoc,* and *Mvk*

**(B-C)** UMAP from (Fig. 5A) annotated with expression of lipid metabolism cluster genes that are highly expressed in human lesional skin granulomas – *Sqle, Ldlr,* and *Marco.*

**Fig. S6.** Statin and cholesterol-deficient diet treatment of mice with severe sarcoidosis reduces granuloma inflammation (see Online Methods for information on sampling)

**(A)** Measurement of wet lung weight of sarcoidosis mice after treatment with cholesterol-deficient diet (CFT) and Atorvastatin/ Lipitor or control diet (n=9) and control DMSO diluent (n= 7).

**(B)** Difference in mouse weight before and after treatment (cholesterol-deficient diet (CFT) and Atorvastatin/ Lipitor or control diet and control DMSO diluent).

**(C)** BODIPY 493/503 neutral lipid expression in F4/80^+^ Mac-2^+^ macrophages in lungs of *TSC2*^KO^ sarcoidosis mice and control (n=5).

**(D)** Expression of CD11b myeloid cell marker expression in lungs of sarcoidosis mice treated with cholesterol-deficient diet (CFT) and Atorvastatin/ Lipitor or control diet and control DMSO diluent.

**MATERIALS & METHODS**

**Study Design & Sampling**

This study was designed to test the role of macrophages in human sarcoidosis granuloma formation and to investigate if macrophages from progressive sarcoidosis patients are genetically predisposed to granuloma formation (elaborated in Results “Spontaneous granuloma formation of macrophages from chronic sarcoidosis patients”). We concentrated on chronic sarcoidosis patients, who represent a critical set of patients with unmet clinical need and few options for therapeutic treatment. To this end, we recruited patients presenting with chronic sarcoidosis at the baseline timepoint of an ongoing interventional clinical trial (EudraCT Number: 2017-004930-27) at the Department of Dermatology in Vienna and included age-matched disease-free volunteers as control group (ECS 2242/2017). Here, we took samples from patients with pulmonary involvement in addition to cutaneous involvement for most critical experiments (Data File S1). The inclusion criteria for the patients in this study were 1) clinical features consistent with sarcoidosis, 2) histological evidence of non-caseating granulomas, and 3) persistent disease- not Löfgren syndrome sarcoidosis. All patients were diagnosed according to the Joint Statement of the American Thoracic Society (ATS), the European Respiratory Society (ERS), and the World Association of Sarcoidosis and other Granulomatous Disorders (WASOG).

We addressed our objectives by 1) observing peripheral blood CD14^+^ monocyte-derived macrophages differentiated with GM-CSF for spontaneous aggregation and formation of *in vitro* granulomas, and 2) performing bulk RNA-Sequencing on these macrophages to find out if the sarcoidosis macrophages are genetically different from healthy controls. We also utilized a progressive sarcoidosis mouse model (see below) and obtained skin biopsies from progressive sarcoidosis patients with cutaneous and pulmonary involvement to validate our findings.

A total of 14 progressive sarcoidosis patients and 10 disease-free controls were included in this study (Data File S1). Data File S1 describes the number of recruited patients and healthy donors, clinical information of the recruited patients, and the number of chronic sarcoidosis patient samples used for the various assays performed in this study. In brief, 5 patients and 5 healthy control samples were used for the macrophage aggregation quantification in Figure 1A-1B, 4 patients samples were used for the immunofluorescence stain in Figure 1C-1F, 5 patients and 4 healthy controls were taken for RNA-Sequencing (Figure 2, Table 1, Data File S1), between 4-6 patient samples (4 for imaging and 6 for FACS) were taken for the *in vitro* drug treatment experiments (Figure 3, Supplementary Figure 3A-3D), and between 3-4 patient samples (images taken in duplicates) taken for tissue biopsy staining (Figure 4; duplicates of 4 patient granuloma biopsy taken for BODIPY 493/503, SREBF-1 and MARCO staining, and duplicates of 3 patient granuloma biopsy taken for LipidTox staining).

For IF staining (Fig. 1C-1F), 5 patient samples were used in total but different patient samples were used for different stainings. For Ki67: RP#5, 12, 24; pS6: RP#2, 5, 11, 24. Therefore, the table lists 5 patients but the graphs depict only 4 and 3 patients. Regarding the IF tissue biopsy stains (in Fig 4, Suppl 3E), the graphs represent duplicates of 4 patients. Again, the table displays the total number of patients used for IF stainings as different patient samples were used for the different stainings. For example, for Bodipy we used patients RP#2, #3, #5, #19, and for SREBF1, patients RP#3, 19, 11, 25.

All patients and control group individuals in this study participated voluntarily with written informed consent in accordance with the Declaration of Helsinki. The study was carried out according to national law and approved by the ethics committee of the Medical University of Vienna (ECS 2242/2017).

Peripheral blood was drawn in BD Vacutainer EDTA blood collection tubes. For sampling of skin, 6 mm punch-biopsies were taken from affected skin and embedded in Tissue-Tek optimal cutting temperature (OCT) compound and stored at -80°C until immunofluorescence staining.

**Blood monocyte isolation**

Peripheral blood mononuclear cells (PBMCs) were isolated from peripheral whole blood under sterile conditions by Ficoll (Pan Biotech GmbH) gradient density centrifugation. CD14^+^ human blood monocytes were collected using anti-human CD14 human MicroBeads (Miltenyi Biotec), as per manufacturer´s protocol.

**Human monocyte-derived macrophage (Mo-Mac) culture**

0.4 x 10^6^ CD14^+^ monocytes per well (Nunclon, 24-well plate) were cultured in VLE RPMI-1640 (Biochrom GmbH) medium supplemented with 10% FCS (Performance Plus, heat inactivated), 1% penicillin-streptomycin (GIBCO), 1% sodium pyruvate (GIBCO) and 1% glutamax (GIBCO) for up to 6 days. Some cells were similarly cultured on ethanol-sterilized glass coverslips and later added to 24-well plates for immunofluorescence staining. CD14^+^ monocytes were differentiated into monocyte-derived macrophages in the presence of 500 IU/mL rhGM-CSF (Immunotools).

***In vitro* granuloma quantification**

For initial cluster in vitro granuloma quantification comparing patient and controls, a manual cluster quantification was performed. On day 6 of culturing, monocyte-derived macrophage cultures were carefully washed and fixed within their respective well with 4% PFA (Biolegend) and stored in PBS at 4°C until further analysis. Light microscopic images of fixed cells were acquired with the Olympus Cell Sens Standard 2.2 software on an Olympus IX51 microscope. The images were imported into visual analysis software ImageJ and macrophage clusters identified in a blinded manner. Cluster size and count was then assessed within ImageJ. To assess changes in cluster count and size before and after treatment, an automated cluster quantification method was used. Here, photomicrographs of DAPI stained cells were taken using the Biotek Cytation 5 or Lionheart FX automated cell imagers (inverted microscopes), and analyzed with the Gen 5 image processing and analysis software.

**Coverslip immunofluorescence staining**

On day 6 of cell culture, coverslips were carefully washed with PBS and fixed with 4% PFA fixation buffer (Biolegend) for 20 minutes. Surface and intracellular staining were performed using directly or indirectly labeled antibodies. In brief, after incubation with the primary antibodies overnight, an appropriate secondary fluorescent-labeled antibody was applied for 1 hour at room temperature, followed by counterstaining with DAPI. Coverslips were then removed from 24-well-plates and mounted on super-frost plus adhesion slides (Thermo Fisher). Immunostainings were controlled with isotype-matched conjugates. Intracellular staining was preceded by a permeabilization step with 0.3% Triton X-100 for 10 minutes at room temperature. For evaluation of immunofluorescence results, images were acquired at room temperature using a Z1 Axio Observer microscope equipped with an LD Plan-Neofluar x20/0.4 objective (Zeiss, Oberkochen, Germany) and quantified using TissueFAXS and/or TissueQUEST image analysis software (Tissue Gnostics, Vienna, Austria).

**BODIPY 493/503 lipid staining for fluorescent microscopy**

After 6 days of culture, cell culture media was removed and cells were gently washed with PBS. 2 μM BODIPY 493/503 (Invitrogen) in PBS was added into the respective wells and incubated at 37°C for 15 minutes, and washed twice with PBS. Cells were then fixed with 4% PFA fixation buffer for 30 minutes, and washed with PBS. Cells were then stored in PBS at 4°C until fluorescent microscopy acquisition with the Biotek Cytation 5 or the Lionheart FX.

***In vitro* granuloma inhibitory assays**

On day 3 of incubation with GM-CSF, all media was removed from the wells and replaced with lipoprotein deficient (LPD) media containing 10% lipoprotein-deficient serum (kindly provided by Herbert Stangl), 1% Penicillin-Streptomycin (GIBCO), 1% glutamax (GIBCO), 1% sodium pyruvate (GIBCO) in VLE RPMI (Biochrom) or standard culture media (10% FCS, 1% Pen-Strep, 1% glutamax, 1% sodium pyruvate in VLE RPMI), and replenished with 500 IU/mL GM-CSF. Thereafter, 5 or 12 μM of Lovastatin (kind gift from Herbert Stangl) or DMSO diluent control was added into the respective well. For Tofacitinib treatment experiments, 400 or 1000 nM Tofacitinib (Selleckchem) or DMSO was added into the respective wells. Cells were cultured for 3 more days under standard cell culture conditions before harvest/ analysis.

**Cholesterol-free diet and Atorvastatin treatment of sarcoidosis mice**

Age-matched male and female *TSC2*^fl/fl^ CD11c Cre + sarcoidosis mice (10) with severe disease that were 45-47 weeks old at the end of the study were fed with cholesterol-free diet (Altromin, C1060) and orally gavaged with 30 mg/kg Atorvastatin/Lipitor (Selleckchem S2077) or with control diet (Altromin, C1000) and vehicle control DMSO diluent for 5 weeks. Mice were treated with control diet or cholesterol-free diet 2 weeks before the 5-week treatment period. The numbers of male (n= 5 each) and female mice (n=4 each) were similarly divided amongst the groups, and age-matched between the groups. 2 female control diet and vehicle-treated sarcoidosis mice died before the end of the treatment period (from one of these mice, we were in time and managed to harvest a lung lobe for histology, measure the spleen length and weigh the mouse). We had technical issues with the FACS samples taken for 2 of the mice treated with control diet, thus there were n=5 for the control diet mouse group in the FACS experiments. All mouse experiments were approved by the Austrian Ethics Committee (GZ.BMBWF 2020-0.547.514) and performed in accordance to institutional and state guidelines.

**Mouse paw thickness and organ measurements**

Mouse paw thickness were taken at the end point of the mouse treatment study, a day after the treatment period. The average of five consecutive measurements of the left hind paw were recorded using a digital thickness gauge (Käfer FD 50/25). The body weight of mice, as well as the wet weight of organs, and length of the mouse spleen was also taken. Murine organs were weighed on a Sartorius Analytical weighing scale (TE1245). To obtain the lung weight index of the mice, the wet weight of the lungs (all lung lobes) was divided by the body weight of the mouse and multiplied by 100.

**Mouse lung histology and granuloma quantification**

The left lung lobe of the mice was excised and fixed overnight in Histofix (4% formaldehyde, Carl Roth). Thereafter the tissue was dehydrated and embedded in paraffin. Lung sections were cut at 2 μm thickness and stained with purified anti-mouse/human Mac-2 (Galectin-3), #CL8942AP from Cedarlane, followed by donkey anti-rat Alexa Fluor 55 (ab150154) secondary antibody and DAPI. Photomicrographs of the stained lung sections were taken with a Z1 Axio Observer microscope equipped with an LD Plan-Neofluar 20x/0.4 objective (Zeiss) and a TissueFAXS imaging system, and analyzed using TissueQuest software (Tissue Gnostics GmbH).

**Flow cytometry**

Skin from the paws and tail (2 or 5 pieces of 1cm x 1cm tail skin) were removed from the mouse and incubated with 1 mg/mL of dispase (Invitrogen) for an hour. Thereafter, epidermal and dermal sheets were further incubated with 0.8mg/mL collagenase IV (Sigma) for two hours. Cells were then vigorously shaked and passed through a 18G syringe and 100μm cell strainer (BD Falcon) to obtain a single cell suspension. Excised murine lung lobes were treated with Collagenase D (1mg/mL), and DNAse I (0.1 mg/mL) for 25 minutes at 37 degrees in a Miltenyi Gentle MACS machine and passed through a 70μm strainer (BD Falcon) to obtain a single cell suspension. Cells were then stained with Fc block (10 minutes) and fluorescent labeled antibodies (20 minutes) at 4 degrees prior to acquisition on a Beckman Coulter Cytoflex S cytometer. For neutral lipid analysis, cells were further stained with Bodipy 493/503 for 15 minutes at 37 degrees Celsius and washed prior to acquisition. Cells were stained with 1μl/mL of 7AAD (Thermo Fisher, A1310) for dead cell exclusion.

Primary human Mo-Macs cultured for 6 days were detached using ice-cold 2mM EDTA (Invitrogen) in PBS, centrifuged, washed, and stained with a fixable live-dead discrimination dye (Zombie NIR, Biolegend). Washed cell pellets were then resuspended in ice-cold methanol (100%) followed by a 20 minute incubation at -20°C. Thereafter, mo-Macs were washed and intranuclear immunostainings performed for 20 minutes at room temperature (RT). Isolated peripheral blood monocytes were fixed with 4% PFA for 10 minutes at RT, centrifuged to remove PFA, and permeabilized in ice cold methanol and placed on ice for 30 minutes or stored at -20 degrees. Cells were then washed with FACS buffer and stained with fluorescent-labeled antibodies for 40 minutes at RT. Alternatively, cells were incubated with Fc block for 10 minutes and cell surface markers stained with fluorescent-labeled antibodies for 20 minutes at 4 degrees. Samples were then washed with PBS and stained with fixable live/dead discrimination dye. Cells were then fixed and permeabilized with eBioscience Foxp3 Transcription Factor staining kit according to manufacturer’s instructions and stained with fluorescent-stained antibodies (list of antibodies found in Supplementary Table) for 20 minutes at RT. Samples were then washed once with PBS and acquired using a FACS Aria III (BD Biosciences) or Beckman Coulter Cytoflex S cytometer and analyzed with FlowJo 10.7.1 Software.

**Cryosection staining**

Punch biopsy samples of human skin were embedded in OCT compound (Tissue-Plus, Scigen Scientific, Gardena, Calif), deep-frozen in liquid nitrogen, and stored at -80 °C until further processing. OCT compound-embedded tissue samples from patients were cut into 7 mm-sections and mounted on super-frost plus adhesion slides (Thermo Fisher Scientific). Air dried sections were fixed in acetone (Sigma Aldrich) for 10 min and stored at -20 °C until immunofluorescence staining. Multi-color immunofluorescence staining for cell surface markers was performed on 7 mm cryosections. Immunofluorescence stainings were performed as previously described (see *Coverslip* immunofluorescence *staining*). For BODIPY 493/503 (Invitrogen) and HCS Deep Red LipidTOX^TM^ (Thermo Fisher Scientific) neutral lipid stainings, cryosections were fixed in 5% neutral buffered formalin solution in PBS for 10 minutes at room temperature. BODIPY 493/503 or LipidTOX dyes were applied for 1 hour at room temperature according to the manufacturer’s protocol. Slides were scanned using a Z1 Axio Observer microscope equipped with an LD Plan-Neofluar 20x/0.4 objective (Zeiss) and a TissueFAXS imaging system and analyzed using TissueQuest software (Tissue Gnostics GmbH). Epidermis, blood vessels and apocrine glands were excluded from analysis. The dermal compartment was subdivided in granuloma areas and areas containing no granuloma structures. Analysis was performed on two representative sections as replicates.

**Bulk RNA-seq data generation**

Up to 1.2 x10^6^ CD14^+^ monocytes or monocyte-derived macrophages from patients and healthy controls were carefully washed, harvested and lysed in 700 ul Qiazol (Qiagen) and stored at -80°C until RNA isolation. CD14^+^ cells were lysed on the day of sample collection, whereas macrophages were harvested after 3 days of differentiation with GM-CSF. RNA was isolated from the aforementioned samples using the miRNeasy Kit (Qiagen) and concentration and purity assessed using a Nanodrop 2000c Spectrophotometer (Thermo Scientific). RNA quality of the samples was further assessed on the Agilent Bioanalyzer RNA 6000 Nano Electropherogram, and library preparation performed on samples with RIN quality between 8-10. 100ng of total RNA was used for library preparation using the NEBNext Ultra II directional RNA library preparation kit with poly-A tail enrichment. Thereafter, stranded mRNA 75 bp single read RNA sequencing was performed on the Illumina NextSeq 500 sequencer.

**Quality control of sequencing reads and read mapping**

Single-end RNA-seq reads were trimmed for low quality bases and removal of low-quality reads using ReadTools (v.1.0.0). Trimmed reads were mapped to the Homo sapiens genome (GRCh38.92 assembly) using STAR (v2.5.3a) mapper. PCR duplicates were eliminated from further analysis by using the MarkDuplicates function from the PICARD package (v 2.22.3; https://broadinstitute.github.io/picard/) and the reads mapped in multiple genomic locations were eliminated by using Samtools (v1.9).

**Bulk RNA-seq analysis**

Read counts for features (exons) were generated using the featureCounts function from the Subread package (v1.5.3). Lowly expressed genes with fewer than 10 read counts in all samples were eliminated from further analysis. Differential gene expression analyses were executed with DESeq2 (v 3.22.3) using the Ensembl Known Gene models (version GRCh38.92) as reference annotations. Genes were considered differentially expressed if they showed a P-value < 0.05 and an absolute log2(fold change) > 1. Normalized expression (log2) by DESeq2 was used to perform the Principal Component Analysis (PCA) and heatmaps.

**Gene Set Enrichment Analyses**

To understand the biological roles of the differentially expressed genes, Enrichr pathway gene-set analyses were performed. Pathways from MSigDB Hallmark 2020, Elsevier Pathway Collection and Biocarta 2016 were utilized. The top 9 significant enriched pathways (according to Enrichr adjusted P-values < 0.05) from the 3 pathway databases were compiled together and ranked. DOSE, org.Hs.eg.db, tidyverse, ggprism, enrichplot (network, heatmaps) packages were used to plot out the figures.

**Protein-protein interaction analyses**

Differentially expressed genes (up or downregulated) between the disease and control groups were converted into proteins using standard STRING settings (https://string-db.org/). Full STRING network analyses with a high minimum required interaction score of 0.970 was performed in confidence mode. Markov Cluster (MCL) Algorithm was used to cluster nodes with an inflation parameter of 3.

**Single-cell RNA-seq data generation and analyses**

40,000 single CD45^+^ expressing cells from paw and tail skin of *TSC2*^fl/fl^ CD11c Cre + sarcoidosis mice and *TSC2*^fl/fl^ controls (n=3 each) were sort-purified. scRNA-seq libraries were generated using the Chromium Controller and the Chromium Single Cell 3' Reagent Kit (v2, 10x Genomics, samples from 2018) and the Next GEM Single Cell 3’ Reagent Kit (v3.1, 10x Genomics, samples from 2021) according to the manufacturer’s instructions. Single cell suspensions obtained from mouse skin were processed individually according to the manufacturer’s protocol. Libraries were sequenced by the Biomedical Sequencing Facility at the CeMM Research Center for Molecular Medicine of the Austrian Academy of Sciences, using the Illumina HiSeq3000/4000 or NovaSeq 6000 platform. Raw sequencing data was pre-processed and demultiplexed using Cell Ranger (v 5.0.1). sc-RNA-seq data were analyzed by Seurat v4.3.0. In Seurat, cowplot was used to normalize data, “JackStrawPlot” and “ElbowPlot” and ‘RunUMAP’ functions were used to identify clusters and principal components. Clusters were further analysed by Seurat default functions.

**Statistical Analyses**

Unless otherwise stated, error bars represent mean ± SEM, and paired Student’s t test or Mann-Whitney test were performed using GraphPad Prism version 5.0 or 9.0 (GraphPad Software Inc).

**Data and materials availability**

The RNA-Sequencing data can be found deposited in the NCBI Geo Database under the accession number GSE251802. The scRNA-seq data are available from NCBI Gene Expression Omnibus under the accession number GSE250508.
