# Supplementary material for: Aberrant lipid metabolism in macrophages is associated with granuloma formation in sarcoidosis
Source: Am J Respir Crit Care Med. Author manuscript; Available in PMC 2025 Mar 21. (PMC7617514; doi:10.1164/rccm.202307-1273OC)

Suppl Figure 1

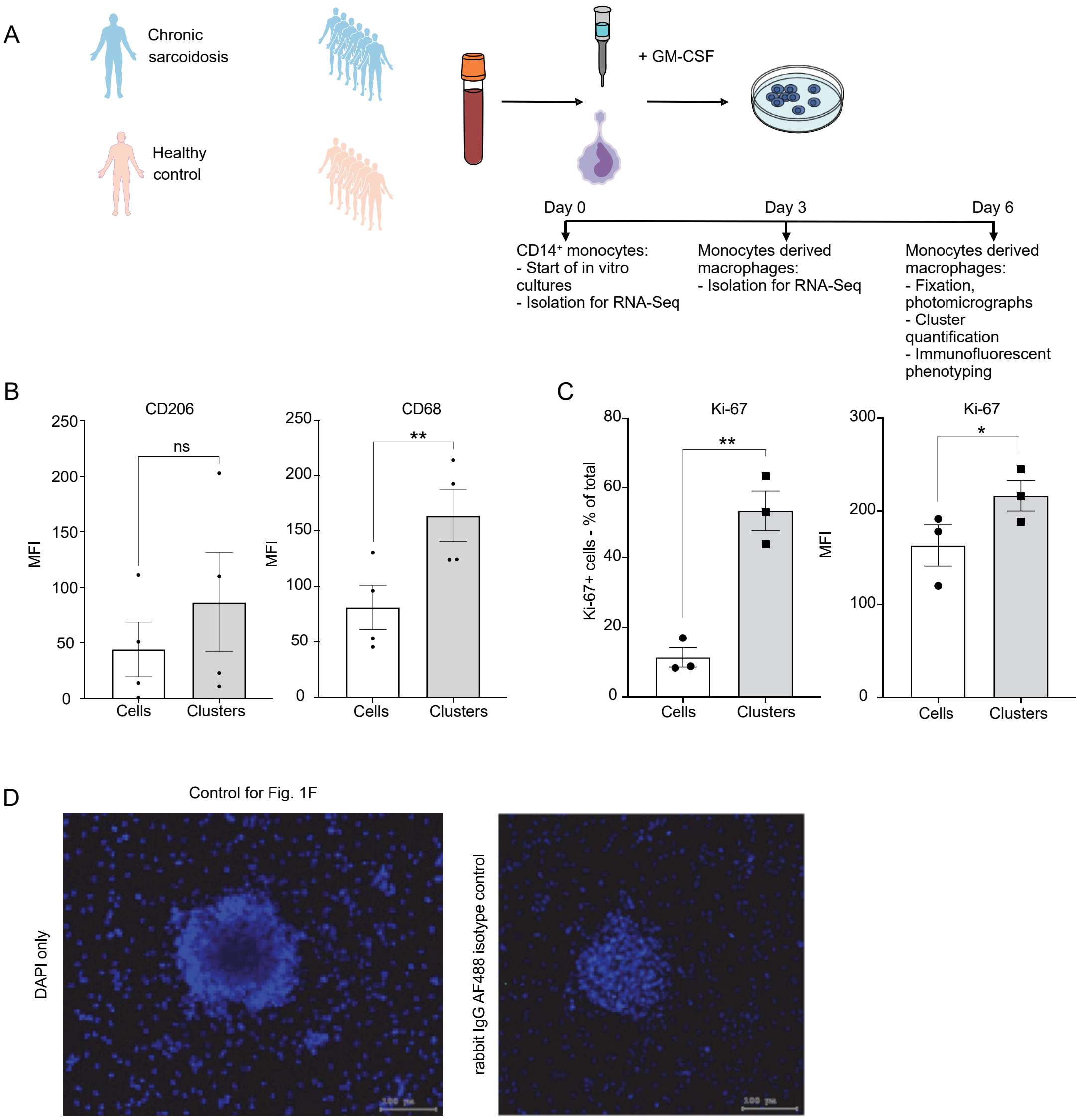

Suppl. Figure 2

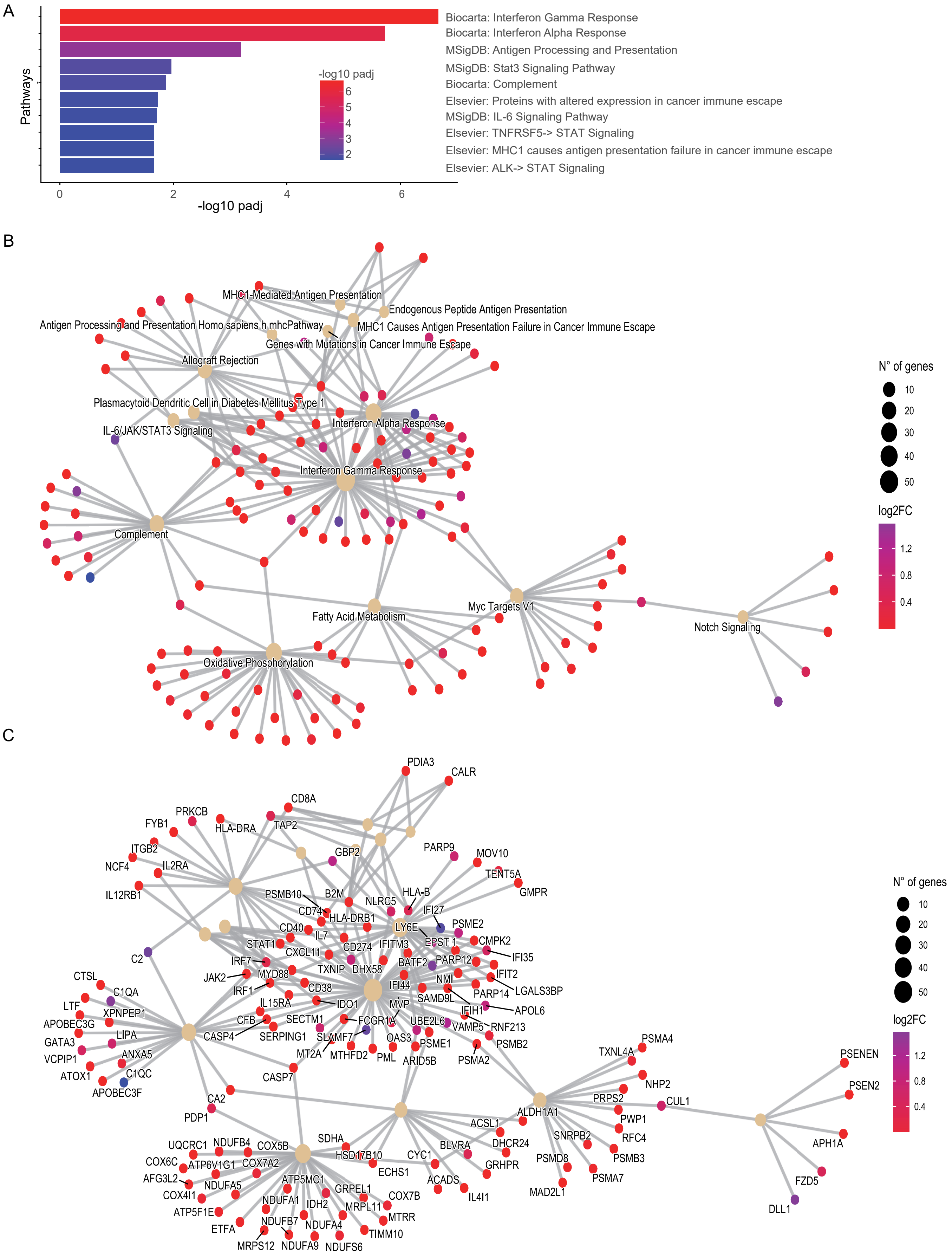

Suppl. Figure 2 (continued)

D

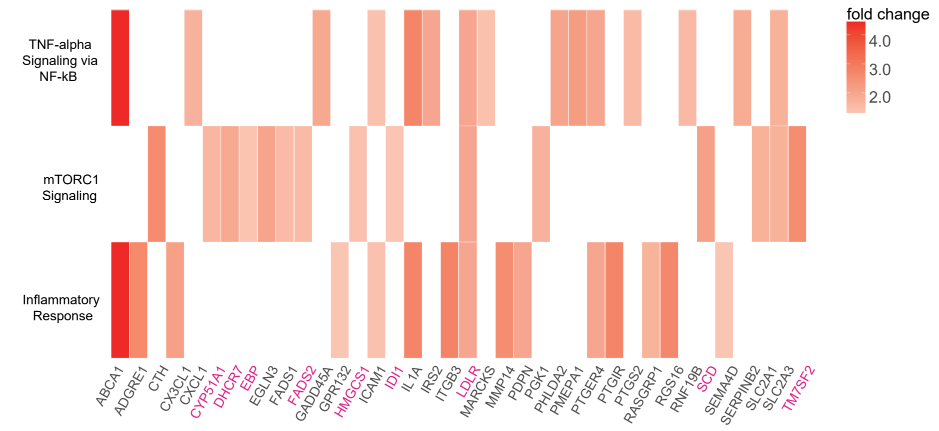

E

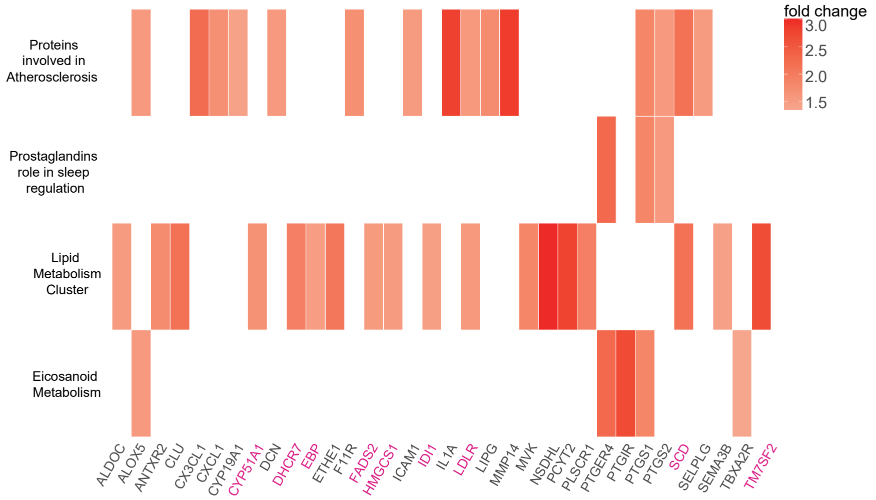

Suppl. Figure 3

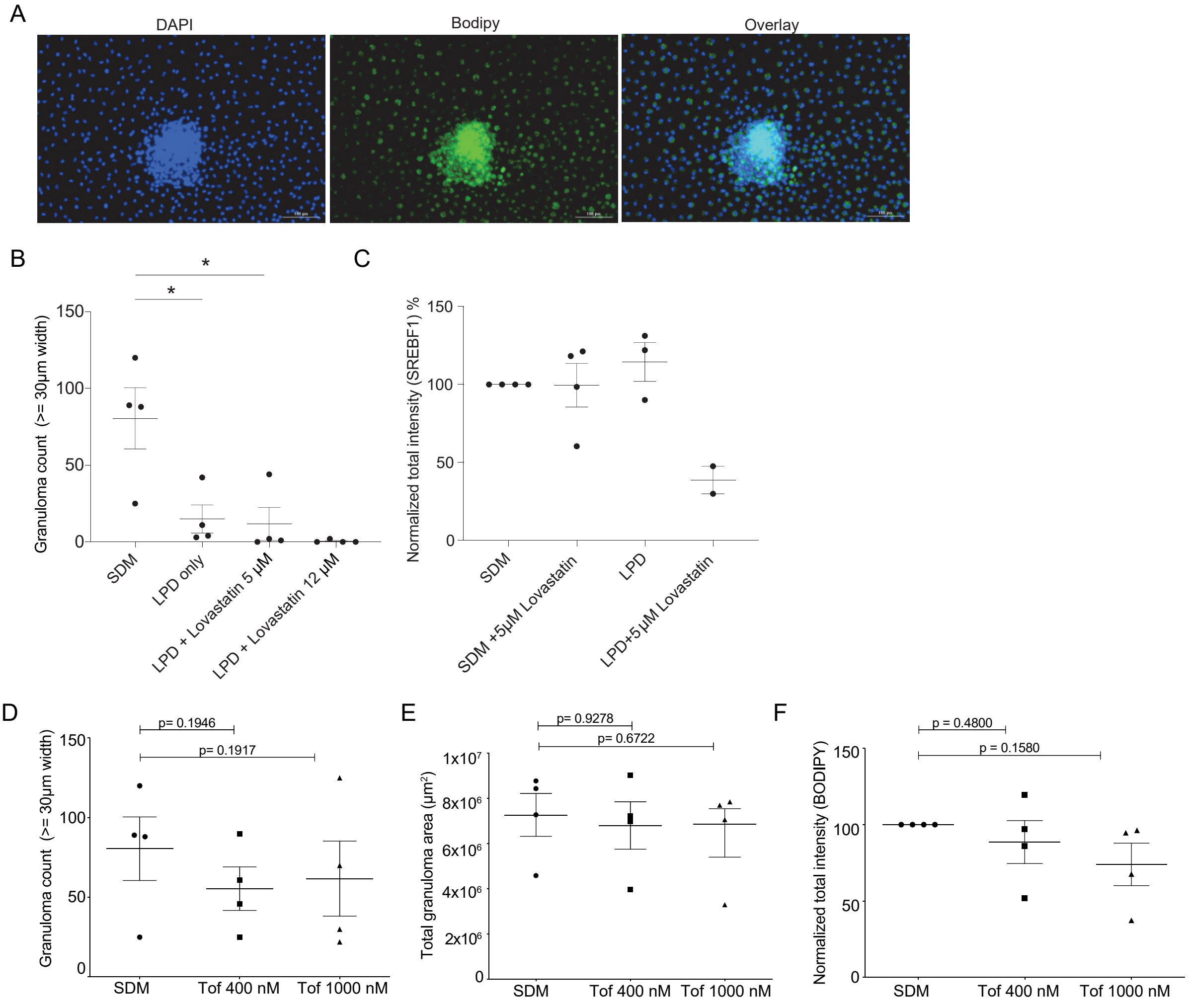

Suppl. Figure 4

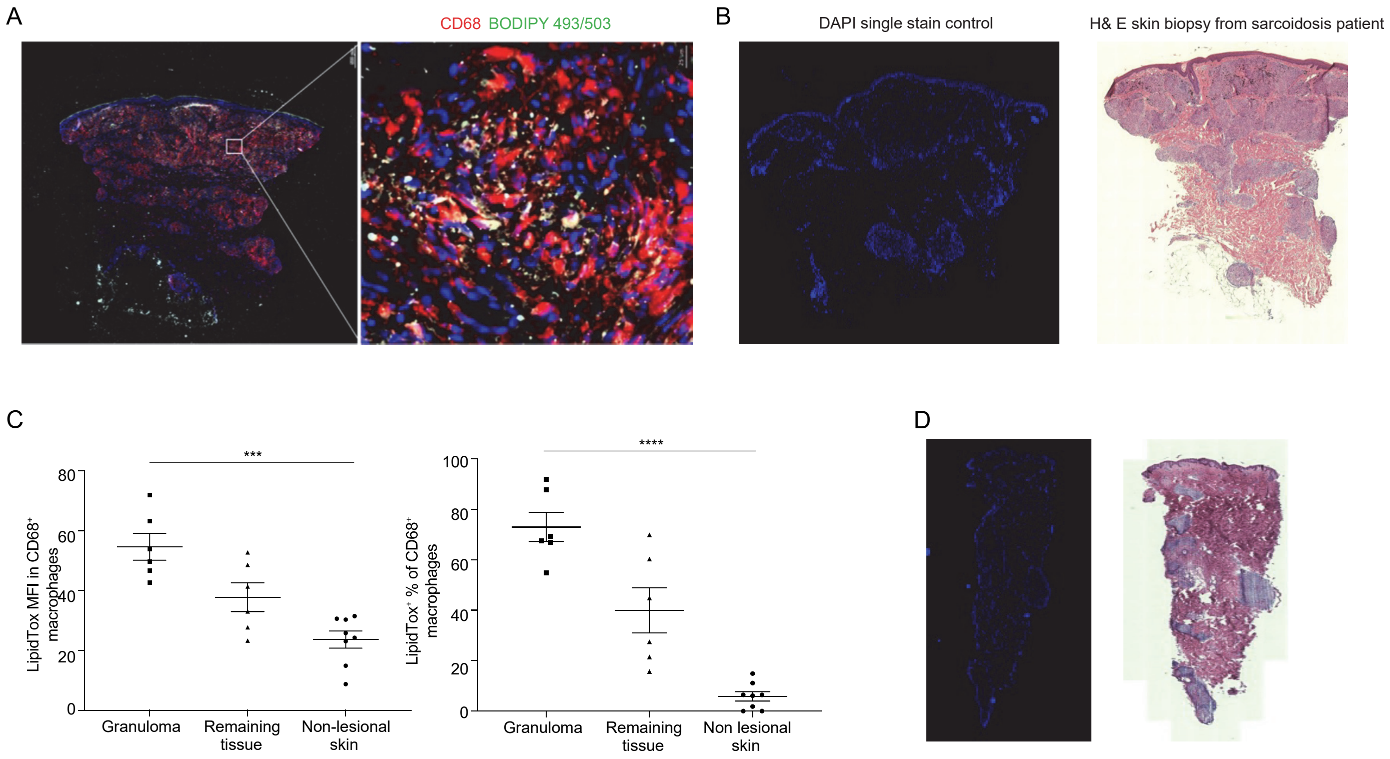

Suppl. Figure 5

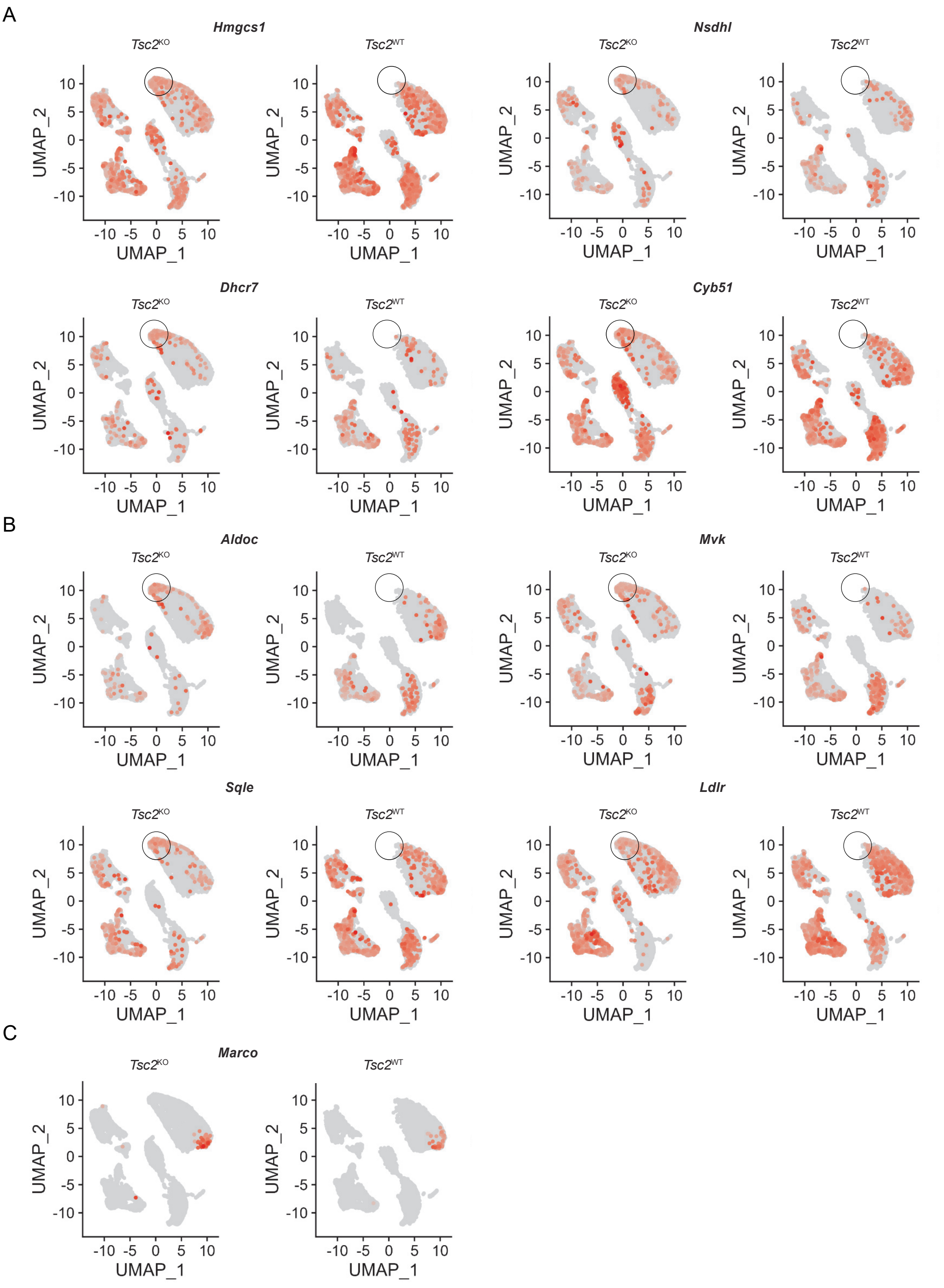

Suppl. Figure 6

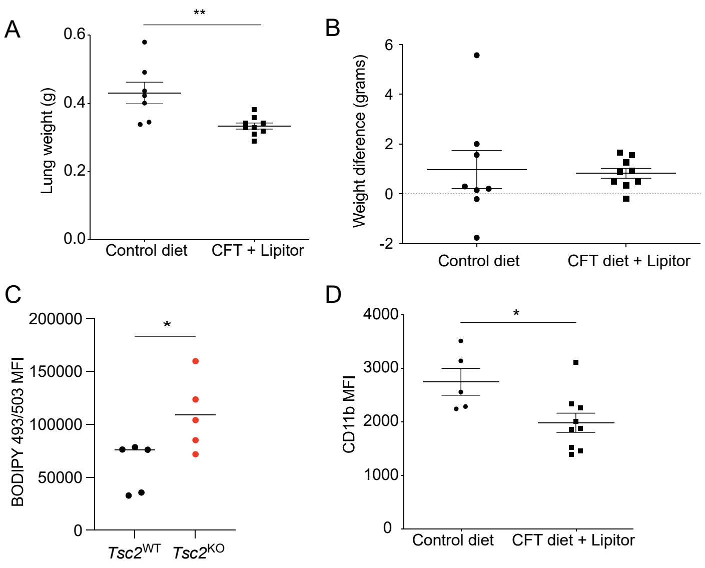

Supplement: Supp. Fig. [file EMS203765-supplement-Supp__Fig_.pdf]
